# Dietary Methionine Affects Lipid Metabolism and Ferroptosis-Related Responses to Modulate Oxidative Stress Induced by High-Lipid-Diet in Golden Pompano (*Trachinotus ovatus*)

**DOI:** 10.3390/antiox15070873

**Published:** 2026-07-14

**Authors:** Bissih Fred, Kaimin Cheng, Agyenim Godfred Boateng, Junming Deng, Beiping Tan, Asare Derrick, Shuyan Chi

**Affiliations:** 1 College of Fisheries, Guangdong Ocean University, Zhanjiang 524088, China; 1252201246@stu.gdou.edu.cn (B.F.); godfred125@stu.gdou.edu.cn (A.G.B.); djunming@163.com (J.D.); bptan@126.com (B.T.); deruike@stu.gdou.edu.cn (A.D.); 2 Aquatic Animals Precision Nutrition and High Efficiency Feed Engineering Research Center of Guangdong Province, Zhanjiang 524088, China; 3 Guangdong Yuehai Feeds Group, Zhanjiang 524000, China; ckmin@yuehaifeed.com

**Keywords:** methionine, lipid metabolism, oxidative stress, *nrf2*/*keap1* pathway, antioxidant defense, *Trachinotus ovatus*, dietary interventions

## Abstract

This study investigated the effects of high-lipid diets (HLD) with methionine (Met) supplementations on golden pompano (*Trachinotus ovatus*). *T. ovatus* (initial weight 82 ± 0.04 g) were fed with a normal lipid control diet (11.30% crude lipid and 1.04% Met, NLM) and HLDs (18% crude lipid) supplemented with varying Met levels (1.04%, 1.14%, 1.24%, 1.34%, 1.44%, 1.54% and 1.64%) namely HLM1, HLM2, HLM3, HLM4, HLM5, HLM6 and HLM7, respectively. After 56 days of feeding trial, the growth performance such as weight gain rate was significantly elevated in the HLM3 (*p* < 0.05). The liver lipid droplets area, sum of n-3 and n-6 poly-unsaturated fatty acids and fatty acid synthesis genes were elevated in the HLM1 and HLM7, while the genes for lipid breakdown were elevated in the HLM3. Based on the groups NLM, HLM1, and HLM3, the transcriptome sequence data revealed critical associated lipid metabolism, classic antioxidant pathway *nrf2* and ferroptosis markers were influenced by Met and higher lipid. The fish livers in HLD groups with lower or higher Met showed the phenomenon of lipid oxidation obviously, while the reactive oxygen species and malondialdehyde were considerably lowered in the liver of fish in group HLM3 (*p* < 0.05). The hepatic Met was significantly reduced while cysteine was elevated in the HLM3 compared to HLM7 (*p* < 0.05). Fe and ferroptosis inducers were significantly upregulated in the liver of HLM1 and HLM7. HLM3 elevated anti-ferroptosis and anti-inflammation markers. In conclusion, Met inclusion in the HLD was associated with *nrf2*/*keap1* and critical anti-ferroptosis-related transcriptional responses and regulated lipid metabolism in *T. ovatus*. The quadratic regression model revealed the optimal dietary Met in the HLD as 1.32%, which will help formulators make more rational and effective use of dietary lipid to support the better growth of golden pompano.

## 1. Introduction

Lipids, carbohydrates, and proteins are crucial nutrients that significantly influence fish metabolism, development, and overall performance [1,2]. As is well known, aquaculture species exhibit a greater demand for dietary protein and amino acids than terrestrial animals [3]. Lipids were found to be more effective than carbohydrates for protein utilization, particularly at lower protein levels in rainbow trout [4]. Especially lipids are recognized for their high energy density, providing approximately 9 kcal/g compared to carbohydrates’ 4 kcal/g [4,5] and then serve as a crucial energy source, facilitating development, immunological function, and reproduction for carnivorous fish with elevated metabolic requirements [6,7,8]. In the last few decades, high-lipid diets (HLDs) have been widely adopted in the intensification of aquaculture production to decrease protein intake and phosphorus and nitrogen emissions, thereby achieving protein savings [9,10,11]. Higher lipid levels had a cascade of negative effects on the physiological tolerance in farmed fish, which were mainly reflected in oxidative stress, inflammatory response, metabolism, and apoptosis and ultimately affected growth in juvenile black seabream (*Acanthopagrus schlegelii*) [12,13] and large yellow croaker (*Larimichthys crocea*) [14]. Research on mitigating the detrimental effects of HLDs in aquaculture has thus become essential.

Methionine (Met) is an indispensable amino acid for protein synthesis and serves as a methyl donor in numerous metabolic pathways, regarded as a crucial dietary regulatory element in lipid metabolism [15]. Suitable dietary Met was proved to decrease lipid accumulation, inflammatory response, and oxidative stress and alleviated liver damage in juvenile black seabream [13]. In rainbow trout (*Oncorhynchus mykiss*), Met restriction inhibits lipogenesis, potentially by down-regulating the gene expression of sterol regulatory element binding protein-1 (*srebp1*) or adenosine monophosphate activated protein kinase (*ampk*) [16]. In addition to its role in lipid metabolism, Met may also influence ferroptosis-related responses through sulfur amino acid metabolism, trans-sulfuration, cysteine availability, glutathione synthesis, and redox regulation [17]. Ferroptosis is an iron-dependent form of regulated cell injury characterized by excessive lipid peroxidation, reactive oxygen species (ROS) accumulation, disrupted iron homeostasis, and impaired glutathione peroxidase 4 (GPX4)-mediated antioxidant defense [18,19]. In fish, accumulating evidence indicates that oxidative stress, lipid peroxidation, and iron overload are closely associated with ferroptosis-related tissue injury and mortality under nutritional or environmental stress [20,21,22].

Golden pompano (*Trachinotus ovatus*, Linnaeus 1758) is a crucial species for the cage aquaculture sector in China and Southeast Asia [23]. These regions account for 75% of worldwide aquaculture production [24,25]. In 2024, China’s domestic aquaculture production of *T. ovatus* attained 305,552 tons, establishing it as one of the top three mariculture species in the nation [26]. HLDs have been widely used to feed *T. ovatus* in practical aquaculture conditions in China; nevertheless, one of the concerns of this approach is inefficient utilization of lipid [8,27]. Given that dietary Met is crucial for protein synthesis and has lipid metabolism enhancing capability, this study investigated the mechanism via which dietary Met mitigates the adverse effects of HLDs in *T. ovatus*.

## 2. Materials and Methods

### 2.1. Experimental Diets

There were eight groups of experimental diets, with crude protein content approximately 43%. The basal diet was named NLM with 11.30% crude lipid and 1.04% Met. The remaining seven diets all contained 18% dietary crude lipid, with added Met of 1.04%, 1.14%, 1.24%, 1.34%, 1.44%, 1.54%, and 1.64%, respectively (HLM1, HLM2, HLM3, HLM4, HLM5, HLM6, and HLM7). All the solid raw ingredients were separately ground into a fine powder, sieved through 60 mesh, weighed accurately based on the formulation (Table 1), and mixed with oils. The expanded pellets with diameter 3.0 mm were made and dried under 25 °C for 72 hrs and stored at −20 °C until administered to fish.

### 2.2. Fish Rearing

The feeding trial was carried out on offshore sea cages (Nansan town, Zhanjiang city, Guangdong Province, China). The experimental fish were purchased from a commercial farm (Sanya, Hainan Province, China) and stocked in sea cages (3.0 × 3.0 × 3.0 m^3^). All fish were fed with a commercial diet (43.98% crude protein and 10.84% crude lipid) for two weeks to adapt to the aquaculture environment. Before the feeding trial, the fish were starved for 24 h and weighed after being anesthetized with eugenol (1:10,000) (Shanghai Reagent Corp., Shanghai, China). *T. ovatus* of similar weight (initial weight 82.34 ± 0.04 g) were distributed into 24 sea cages (1.0 × 1.0 × 1.5 m^3^) at a density of 40 fish per cage. Each experimental diet was randomly administered to three cages. The feeding amount was set at 2% of the body weight at the beginning of the experiment, and then the subsequent feeding amounts were adjusted according to the above-mentioned feeding situation until the fish reached apparent satiety. The fish were hand-fed the experimental diets at 8:00 a.m. and 5:00 p.m. over a period of 56 days. During the feeding trial, the sea water quality parameters were temperature 28.8 ± 0.55 °C, salinity 25 ± 0.58, dissolved oxygen 6 ± 0.88 mg/L, and pH value 8.0 ± 0.40. The fish were cultured under natural light and dark conditions.

### 2.3. Sample Collection and Analysis

After 56 days’ feeding period, all the experimental fish in each experimental net were starved for 24 h, and counting and bulk weighing the *T. ovatus* in each replicate were done to ascertain survival rate, specific growth rate, and weight gain rate. The body weight, length, liver weight, and visceral weight of three randomly selected *T. ovatus* from each replication were measured to evaluate the morphological indicators. Nine fish from each replicate were randomly selected for whole fish body composition analysis. *T. ovatus* livers were sampled from 6 fish in every replicate and later used for amino acid profile and fatty acid profile tests. The methods of nutrition and composition analysis for each treatment diet and whole fish were dried at 105 °C to constant weight for moisture detection, Dumas combustion method for protein, Soxhlet extraction using petroleum ether method for crude lipid content and combusted to constant weight at 550 °C for crude ash. The amino acid profile was assessed according to the Chinese standard GB/T 18246-2019 [28] (Table 2), while the fatty acid profile was analyzed according to the Chinese standard GB 5009.168-2016 [29] (Table 3).

Blood was drawn from the caudal vein of 6 randomly chosen fish from each triplicate cage using a syringe without heparin. The blood samples were then centrifuged at 1252× *g* for 10 min at 4 °C. The serum was separated and analyzed using test kits (Nanjing Jiancheng Bioengineering Institute, Nanjing, China) for triglyceride (TG, F001-1-1), total cholesterol (T-CHO, A11-1-1), the contents of high-density lipoprotein cholesterol (HDL-C, A112-2-1), low-density lipoprotein cholesterol (LDL-C, A113-1-1), alanine transaminase (ALT, C009-2-1), nonesterified fatty acid (NEFA, A042-1-1) and aspartate aminotransferase (AST, C010-2-1). Serum biochemical indices were measured using an automatic biochemical analyzer (Hitachi 7020, Hitachi Science Systems, Hitachinaka, Japan). After sampling, fish were dissected on ice, and liver tissues were collected from three fish per cage. The liver samples were placed in RNase-free tubes, immediately frozen in liquid nitrogen, and stored at −80 °C for subsequent analysis of antioxidant status and enzyme activities. Commercial assay kits from Nanjing Jiancheng Bioengineering Institute (Nanjing, China) were used to determine glutathione peroxidase (GSH-Px, A005-1-2), malondialdehyde (MDA, A003-1-2), superoxide dismutase (SOD, A001-3-2), total antioxidant capacity (T-AOC, A015-2-1), catalase (CAT, A007-1-1), and immunoglobulin M (IgM, E025-1-1) in both serum and liver samples. Hepatic iron content was measured using a tissue iron assay kit (Fe, A039-2-1; Nanjing Jiancheng Bioengineering Institute, Nanjing, China). Hepatic ROS levels were determined using an ELISA kit (ROS, YJ266025) according to the manufacturer’s instructions (Shanghai Enzyme-linked Biotechnology Co., Ltd., Shanghai, China).

The liver samples for histomorphological analysis stained by hematoxylin and eosin (H&E) and oil red o were tested by Wuhan Service Biotechnology Co. Ltd. (Wuhan, China) after liver samples were collected from three fish per cage, fixed in 4% paraformaldehyde, dehydrated, embedded in paraffin, and sectioned at 4–5 μm. For oil red o staining, fresh liver tissues were embedded in optimal cutting temperature compound, frozen, sectioned, stained with oil red o, and counterstained with hematoxylin to evaluate hepatic lipid accumulation.

For each treatment, liver sections from nine fish were examined. Three sections per fish and five non-overlapping microscopic fields per section were captured at the same magnification. Oil red o-positive areas were quantified using ImageJ software (version 1.54r, National Institutes of Health, Bethesda, MD, USA) and expressed as the percentage of lipid-positive area relative to the total tissue area. The same thresholding criteria were applied to all images. Representative micrographs were selected based on their consistency with the average histological appearance and quantitative lipid-area results of each group. Image selection and quantification were performed in a blinded manner.

### 2.4. Transcriptome Sequencing and Real-Time Quantitative PCR (RT-qPCR)

For transcriptomic analysis, liver samples were selected from three representative groups: NLM, HLM1, and HLM3. These groups represented the normal-lipid control, high-lipid diet with insufficient Met, and high-lipid diet with optimal Met supplementation, respectively. Three biological replicates were analyzed per group, giving a total of nine RNA-seq libraries. Total RNA was extracted from liver tissues, and RNA quality was assessed using agarose gel electrophoresis, NanoPhotometer spectrophotometry, Qubit 2.0 Fluorometer (Life Technologies, Carlsbad, CA, USA) and Agilent 2100 Bioanalyzer (Agilent Technologies, Santa Clara, CA, USA). Eukaryotic mRNA was enriched using Oligo(dT) magnetic beads, fragmented, reverse-transcribed into cDNA, ligated with sequencing adapters, amplified by PCR, and purified to construct paired-end RNA-seq libraries.

Raw reads were filtered using fastp (version 0.23.4) to remove adapters, reads with >10% unknown bases, poly(A) reads, and low-quality reads. After filtering, 45,179,512–51,445,340 clean reads were obtained per sample, corresponding to 6.73–7.67 Gb clean data, with Q20 values of 97.00–97.59%, Q30 values of 92.24–93.43%, and GC contents of 49.96–50.85%. Residual rRNA reads were removed using Bowtie2 (version 2.2.8), and the remaining reads were mapped to the *Trachinotus ovatus* reference genome using HISAT2 (version 2.1.0; default parameters), with total mapping rates of 90.78–93.62% and unique mapping rates of 85.01–87.87%.

Transcript reconstruction and expression quantification were performed using StringTie (version 1.3.4; -f 0.3) and RSEM (version 1.2.19), respectively, and expression levels were calculated as read counts and TPM. Differentially expressed genes were identified using DESeq2 (version 1.20.0) based on raw read counts. Multiple-testing correction was performed using false discovery rate (FDR), and genes with *p* < 0.05 and |log_2_FC| > log_2_(1.5) were considered significantly differentially expressed. GO and KEGG enrichment analyses were performed using the hypergeometric test. The raw RNA-seq reads have been deposited in the NCBI Sequence Read Archive under BioProject accession number PRJNA1482782.

A total of 10 key genes were selected for RT-qPCR analysis. AIDpure total RNA kit 010-82796972 (Aidlab Biotech Co Ltd., Beijing, China) was used to extract total RNA from the liver samples (approximately 300 mg). A Prime Script RT kit (TaKaRa, Osaka, Japan) was used to synthesize cDNA. The RT-qPCR was performed with a SYBR Premix Ex Taq kit (TaKaRa, Osaka, Japan). A quantitative thermocycler (Light Cycler 480II, Roche Diagnostics, Basel, Switzerland) was performed. Conditions were cycled (95 °C and 30 s), followed by 35 cycles (95 °C and 5 s, 60 °C and 25 s, 72 °C and 30 s). In the relative expression analysis, the β-actin was used as an internal control and the 2^−ΔΔCt^ method was used to calculate the results. According to the transcriptome sequencing data, the target genes’ primer sequences are outlined in Table S1 and all the primers were synthesized by Sangon Biotech (Shanghai) Co., Ltd., Shanghai, China.

### 2.5. Calculation Formula

Weight gain rate (WGR, %) = 100 × (final body weight (FBW, g) − initial body weight (IBW, g))/initial body weight (IBW, g)

Specific growth rate (SGR, %/days) = 100 × [*ln*(final weight (g)) − *ln*(initial weight (g))]/days

Feed efficiency (FE) = 100 × (weight gain (g)/feed intake (g))

Protein efficiency ratio (PER) = FBW (g) − IBW (g)/total protein intake (g).

Lipid efficiency ratio (LER) = FBW (g) − IBW (g)/total lipid intake (g).

Condition factor (CF, g/cm^3^) = 100 × body weight (g)/(the body length (cm^3^))

Hepatopancreas index (HSI; %) = 100 × hepatic weight (g)/body weight (g)

Viscerosomatic index (VSI; %) = 100 × visceral weight (g)/body weight (g)

### 2.6. Statistical Analysis

The one-way analysis of variance (ANOVA) was used to analyze all data, and significant differences between dietary groups were estimated using Tukey’s multiple comparison test, IBM SPSS Statistics, version 27.0.1.0 (IBM Corp., Armonk, NY, USA). The experimental results are presented as mean ± standard error (SE). The significant difference was established at *p* < 0.05. Results were visualized utilizing GraphPad Prism 10. The circle-base triangle correlogram heatmap showing the Pearson correlation between the various key indices was plotted on the online platform https://www.bioinformatics.com.cn (accessed on 4 January 2026).

## 3. Results

### 3.1. Growth Performance of Trachinotus ovatus

**Figure 1:**
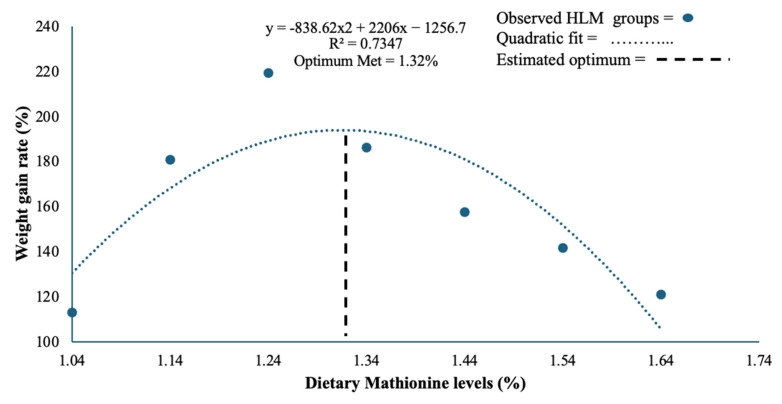
Quadratic regression analysis between dietary Met inclusion level and weight gain rate (WGR) of golden pompano fed HLDs.

At the end of the 56 days of the feeding trial, the FBW, SGR, and WGR were markedly increased in HLM3 (*p* < 0.05); also, the FE (Table 4) was notably elevated in HLM3 compared to the HLM1, HLM6, and HLM7 groups (*p* < 0.05). The HSI was elevated in HLM3 and HLM4 compared to the other groups (*p* < 0.05). The PER and LER were significantly elevated in the HLM3 group but reduced in HLM1 and HLM7 (*p* < 0.05). The VSI and CF did not show significant differences across all the groups (*p* > 0.05). As shown in Figure 1, quadratic regression analysis of WGR against dietary Met level in the HLD groups indicated that WGR increased with increasing dietary Met level up to an estimated optimum and then declined at higher inclusion levels. The fitted regression equation was *y* = −838.62 *x*^2^ + 2206*x* − 1256.7, with *R*^2^ = 0.7347, where *y* represents WGR (%) and *x* represents dietary Met level (% dry matter). The quadratic regression model estimated the optimal dietary Met requirement to be 1.32% that made fish fed HLD get better WGR.

### 3.2. Fish Whole Body Composition

After the 56 days of the feeding trial (Table 5), the crude lipid of whole fish was considerably increased in the HLM1, HLM4, HLM5, HLM6, and HLM7 groups compared to the NLM, HLM2 and HLM3 groups (*p* < 0.05). The crude protein of the whole body of the experimental fish was significantly lower in the HLM1 and NLM groups compared to the remaining groups (*p* < 0.05); however, the whole fish’s body moisture content was not statistically significantly different across all the experimental groups (*p* > 0.05).

### 3.3. Serum and Liver Biochemical Indices and Antioxidant Defense Parameters Across Dietary Treatment Groups

The serum TG and T-CHO contents in groups HLM1 and HLM7 were markedly increased compared to NLM, HLM2 and HLM3 (*p* < 0.05) (Table 6). The HDL-C levels were elevated in NLM, HLM2 and HLM3 compared to the other groups (*p* < 0.05). Comparative to the other groups, the serum LDL-C levels were significantly decreased in the NLM, HLM2 and HLM3 groups (*p* < 0.05). The serum AST and ALT contents were significantly elevated in the HLM1, HLM5, HLM6, and HLM7 groups (*p* < 0.05) compared to the remaining groups.

The effect of dietary Met inclusion in HLDs on the serum and hepatic IgM and antioxidative parameters of adult *T. ovatus* are listed in Table 7 and Table 8. The SOD activity in the fish serum and liver of groups NLM, HLM2, and HLM3 was significantly increased compared to HLM1 and HLM7 (*p* < 0.05). The activities of CAT and GSH-px in the serum and liver were significantly decreased in HLM1, HLM6 and HLM7 compared to the HLM3 (*p* < 0.05). The IgM of fish in groups NLM and HLM3 was increased compared to HLM1 and HLM5-HLM7 (*p* < 0.05). The serum and liver MDA content in groups NLM, HLM2, and HLM3 was lower than that in HLM1 and HLM4-HLM7 (*p* < 0.05). The T-AOC was increased in NLM, HLM2, and HLM3 and significantly decreased in HLM1, HLM6, and HLM7 (*p* < 0.05) in both liver and serum.

### 3.4. Hepatic Met, Cysteine, and Fatty Acid Profile of T. ovatus

**Figure 2:**
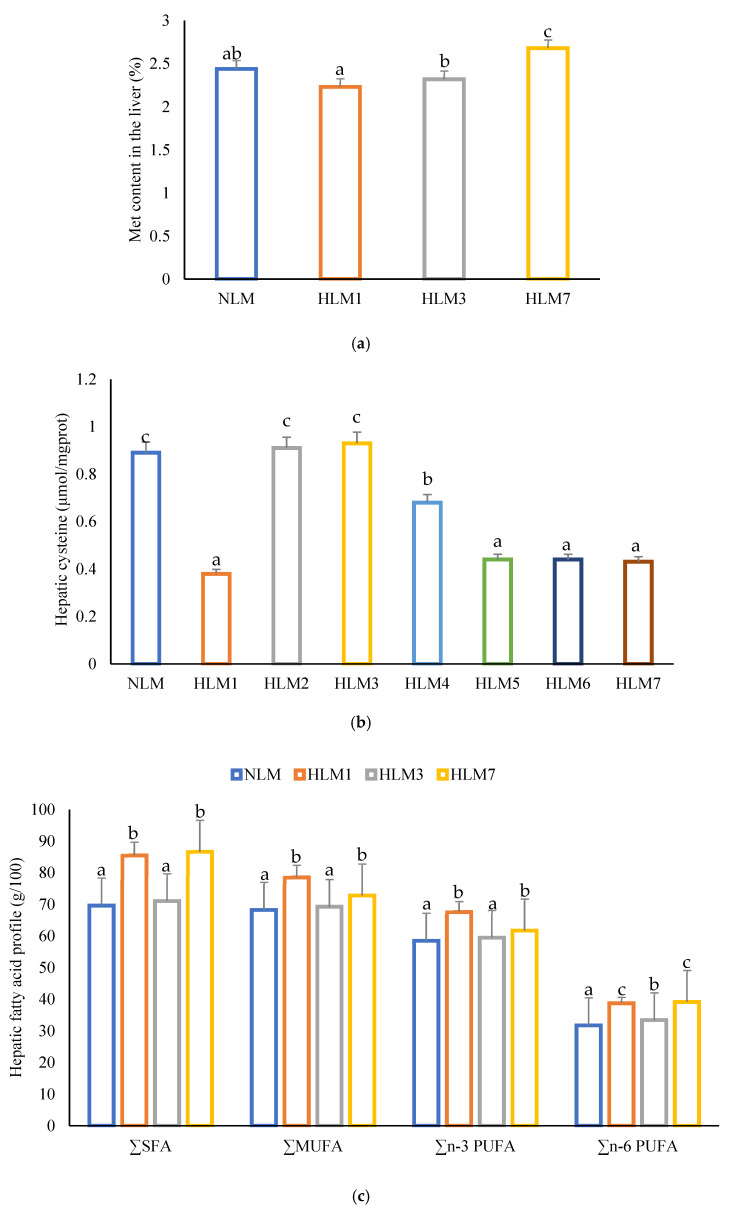
Effect of ingested high-lipid diet and Met on liver contents. (**a**) Hepatic Met content, (**b**) liver cysteine content, and (**c**) fatty acid profile in the liver of *T. ovatus.* Different letters indicate significant differences among groups (*p* < 0.05), as determined by one-way ANOVA. The same notation is used for the data presented in the other figures.

The hepatic Met content in HLM7 group was significantly elevated compared to HLM1 and HLM3 groups (*p* < 0.05) (Figure 2a). The cysteine content in the liver was significantly increased in the HLM2 and HLM3 groups compared to the other groups (*p* < 0.05) (Figure 2b). The ∑SFA, ∑MUFA, ∑n-3 PUFA, and ∑n-6 PUFA were significantly increased in the HLM1 and HLM7 groups compared to the NLM and HLM3 groups (*p* < 0.05) (Figure 2c).

### 3.5. Lipid Accumulation in the Liver of T. ovatus

**Figure 3:**
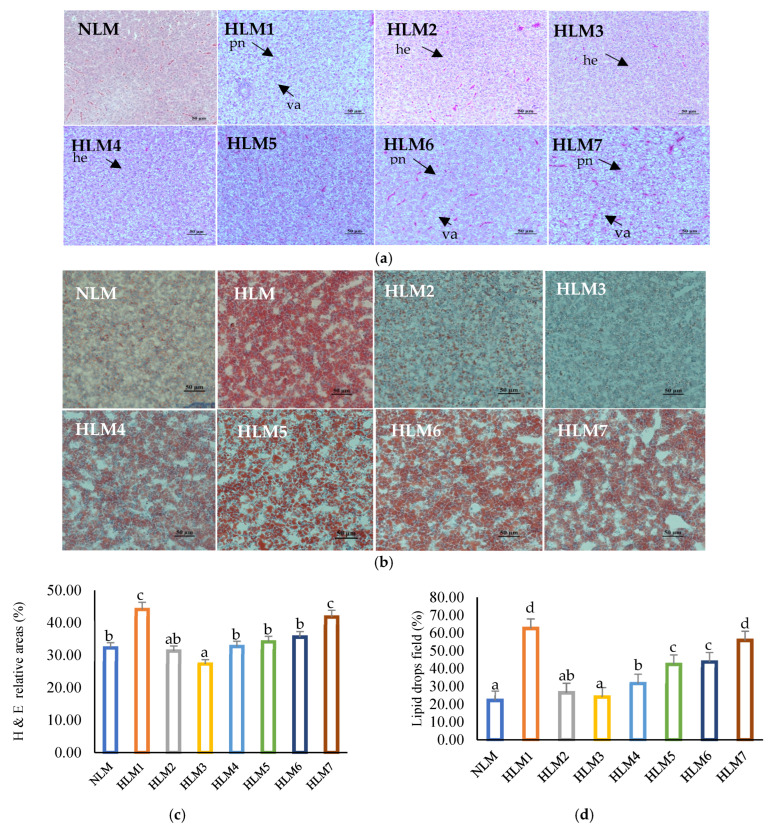
Effects of dietary Met and 18% high-lipid diet on lipid accumulation in the liver of adult *T. ovatus*. (**a**) Histology slice of liver with hematoxylin and eosin staining (×400). (**b**) The oil red O staining (×400) of experimental fish liver. (**c**) Relative area in the liver of the H & E staining. (**d**) Hepatic oil red O staining lipid droplets field. Pyknotic nuclei (pn), vacuoles (va), and hepatocytes (he).

The results of the relative lipid droplet area in the H&E staining showed that 1.24% dietary Met (HLM3) significantly reduced lipid droplets area (27.34%), while 1.02% and 1.63% dietary Met groups significantly increased the lipid droplets field 44.17% (HLM1) and 41.83% (HLM7), respectively (*p* < 0.05) (Figure 3a,c). Dietary Met of 1.02% and 1.63% markedly increased the lipid droplet field 62.55% (HLM1) and 55.84% (HLM7), respectively, compared to NLM (*p* < 0.05), which is based on oil red O staining (Figure 3b,d).

### 3.6. Hepatic Transcriptome Sequence of T. ovatus

#### GO and KEGG Enrichment Analysis of DEGs

**Figure 4:**
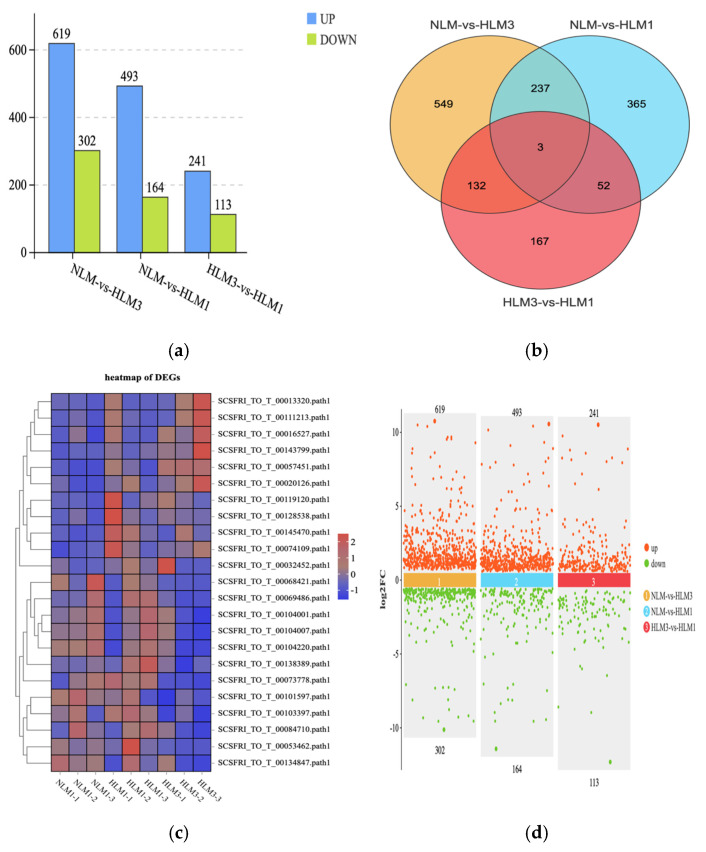
Effect of dietary Met and high-lipid on the transcriptome sequence statistics of *T. ovatus*. (**a**) Bar graph of differentially expressed genes (DEGs) between *T. ovatus* fed with NLM, HLM1, and HLM3. (**b**) Venn diagram of DEGs of NLM, HLM1 and HLM3. (**c**) Heatmap of differentially expressed genes (DEGs). (**d**) Scatter graph of differentially expressed genes (DEGs).

**Figure 5:**
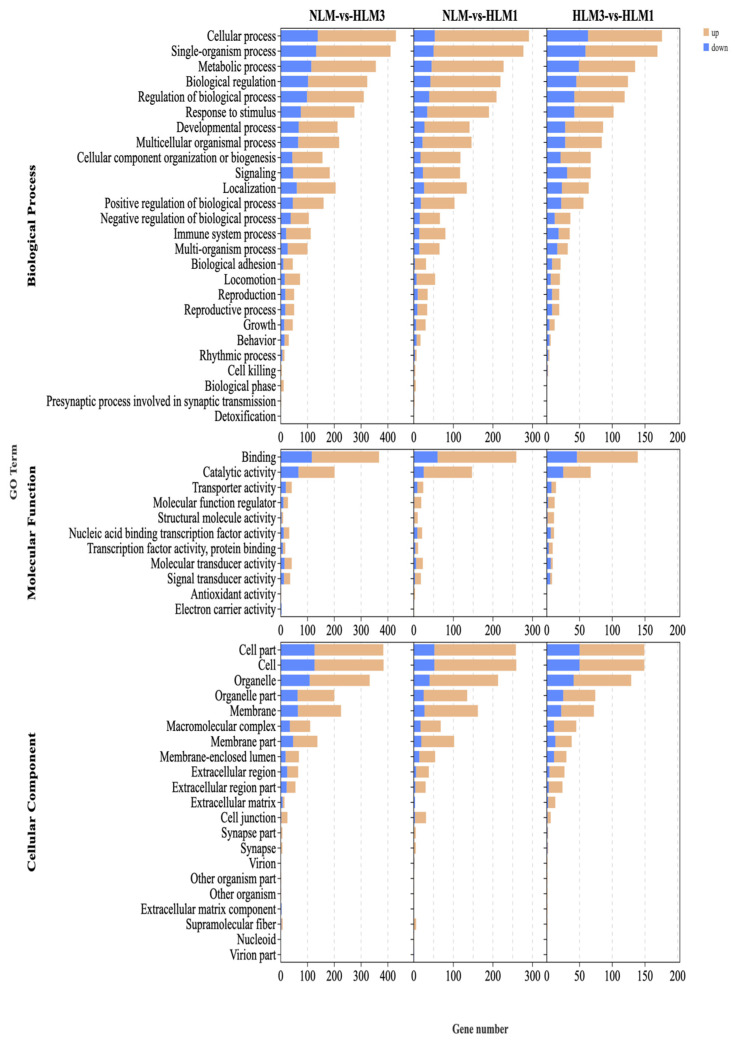
GO enrichment analysis of DEGs in *T. ovatus* fed with NLM, HLM1 and HLM3. Three main GO categories: cellular component, molecular function, and biological process. *X*-axis indicates the number of DEGs, and *Y*-axis indicates GO categories and subcategories.

The GO database was used to further annotate the DEGs and evaluate their functionality. GO analysis showed that DEGs were higher in the NLM, HLM1, and HLM3 groups (Figure 4a–d). All DEGs were classified into three broad functional classes (cellular components, molecular functions, and biological processes), with 58 subcategories (Figure 5). There were 26 significantly enriched terms in the biological process (BP), cellular process category, which included reaction to stimuli, biological regulation, single-organism process, metabolic process, signaling, localization, growth, detoxification, and others. Another 11 significantly enriched phrases were classified as molecular function (MF), which included antioxidant activity, binding, molecular transducer activity, structural molecule activity, catalytic activity, signal transducer activity, transcription factor activity, and so on. The remaining 21 highly enriched terms belonged to the cellular component (CC), such as cell portion, macromolecular complex, extracellular area, supramolecular fiber, membrane- enclosed lumen, and so on. The cell process involved the most DEGs, followed by cell portion, catalytic activity, metabolic activities, single organism processes, binding, and biological regulators.

**Figure 6:**
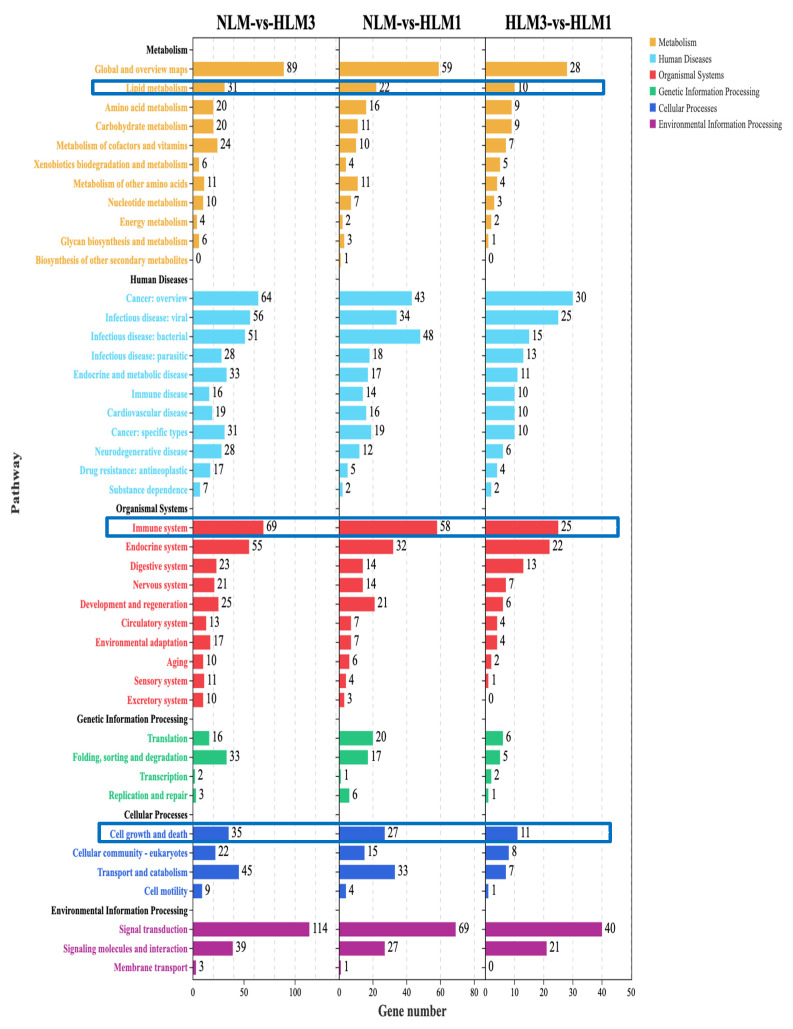
KEGG enrichment analysis of DEGs of *T. ovatus* fed with NLM, HLM1 and HLM3 dietary treatments. Highly expressed biological pathways were represented in the transcriptome retrieved from the KEGG database. DEGs were assigned to six special KEGG pathways, including organismal systems, metabolism, genetic information processing, environmental information processing, cellular processes, and human diseases.

As shown in Figure 6, KEGG enrichment analysis revealed that differentially expressed genes in the NLM vs. HLM3 and HLM3 vs. HLM1 comparisons were mainly distributed across the six major KEGG.

**Figure 7:**
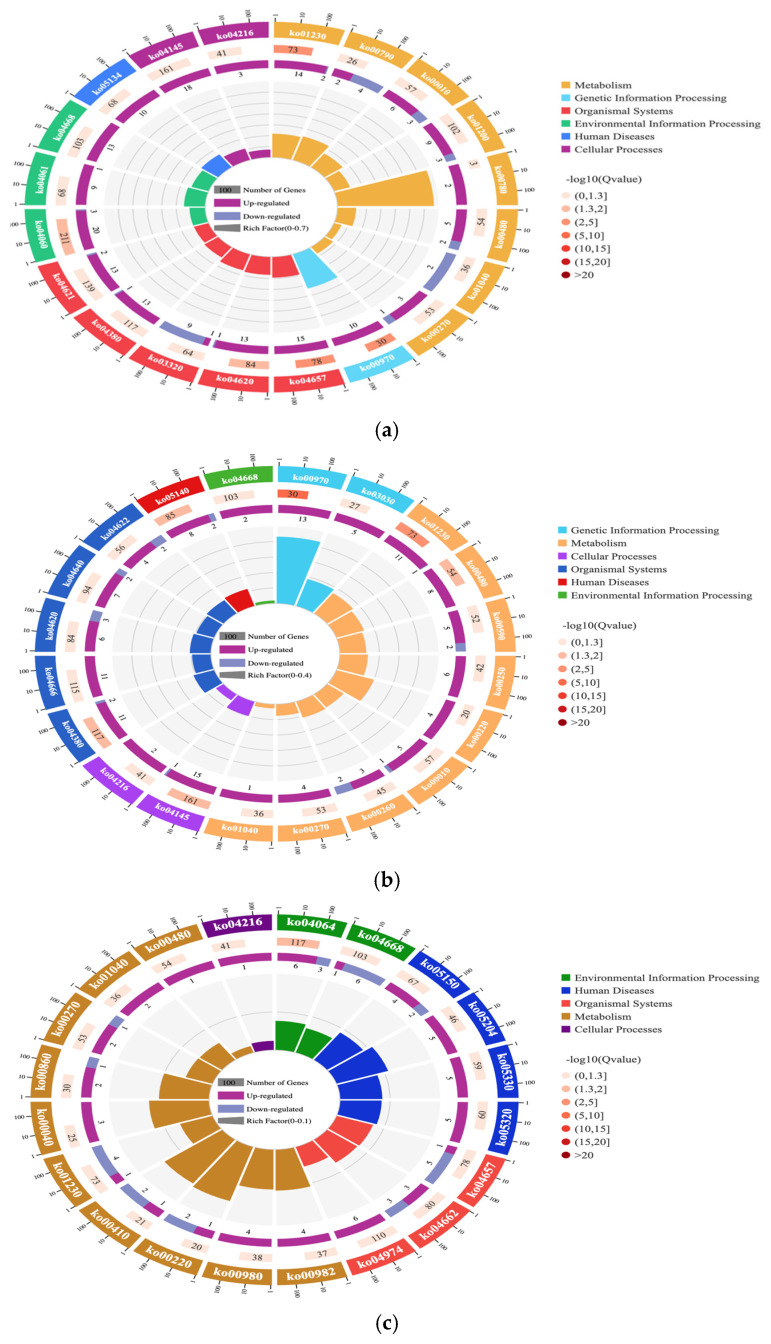
KEGG pathway enrichment analysis showing significantly enriched pathways, where (**a**) represents NLM vs. HLM3, (**b**) HLM3 vs. HLM1, and (**c**) NLM vs. HLM1.

KEGG enrichment analysis revealed that differentially expressed genes between the NLM-vs-HLM1 groups were associated with environmental information processing, metabolomics, human disease, organismal systems, and cellular processes (Figure 7a–c). The DEGs between all the group combinations were involved in *mTOR* signaling pathway (ko04150), arginine biosynthesis (Ko00220), beta alanine metabolism (ko00410), biosynthesis of amino acids (ko01230), pentose and glucuronate interconversions (ko0040), porphyrin metabolism (ko00860), ferroptosis (ko04216), cysteine and Met metabolism (ko00480), biosynthesis of unsaturated fatty acids (ko01040) and *PI3K-Akt* signaling pathway (ko04151).

**Figure 8:**
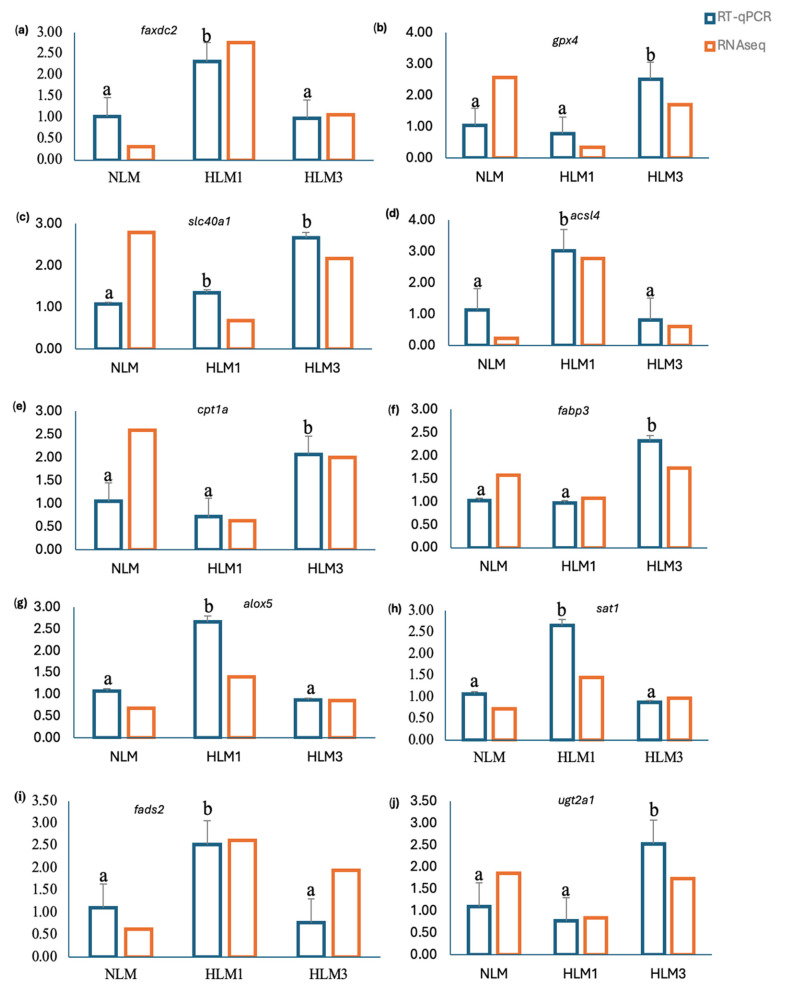
(**a**–**j**) Validation of DEGs associated with the ferroptosis (ko04216) and lipid metabolism pathways by qPCR. The detection of gene expression was done in triplicate for each sample. Expression levels were normalized to those of β-actin using the Livak (2^−ΔΔCt^) technique, and the data are reported as the means ± SD of triplicate tests.

For the purpose of our study, 10 DEGs (*faxdc2*, *gpx4*, *slc40a1*, *acsl4*, *cpt1a*, *fabp3*, *alox5*, *sat1*, *fads2*, and *ugt2a1*) associated with ferroptosis (ko04216) and biosynthesis of unsaturated fatty acids and thus lipid metabolism (ko01040) pathways were selected from the transcriptome data and validated via RT-qPCR (Figure 8a–j).

### 3.7. Lipid Metabolism in the Liver of T. ovatus

**Figure 9:**
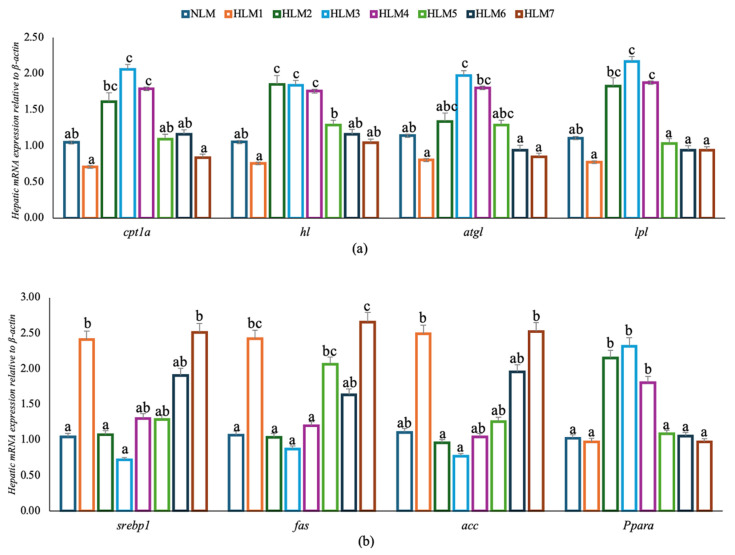
Effect of dietary Met on relative mRNA expressions of lipid metabolism genes. (**a**) Relative gene expression involved in lipolysis in the liver of experimental fish. (**b**) Relative hepatic mRNA expression involved in lipogenesis and *Pparα*.

The hepatic expression levels of genes related to lipid metabolism pathways of adult *T. ovatus* are represented in Figure 9. The experimental fish fed with HLM3 markedly increased the expression levels of the genes cpt1a, hl, atgl, lpl, and Pparα involved in lipolysis; meanwhile, the HLM1 and HLM7 experienced lowered expression levels (*p* < 0.05) (Figure 9a). With regard to the lipogenesis pathway, the mRNA expression levels of the genes srebp1, fas, and acc were highly expressed in the groups HLM1 and HLM6 (*p* < 0.05), yet significantly lowered in the HLM2 and HLM3 (*p* < 0.05) (Figure 9b).

### 3.8. Inflammatory Response in the Liver of T. ovatus

**Figure 10:**
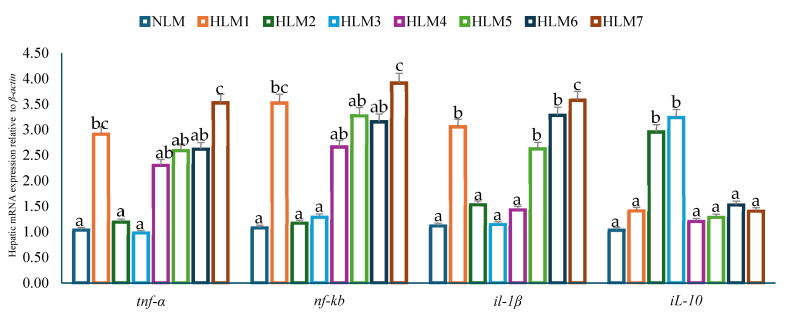
Effect of dietary Met and high lipid on the hepatic mRNA expression of inflammatory-response-related genes.

The mRNA expression of inflammation-related genes are shown in (Figure 10). The expression levels of the pro-inflammation-related genes *tnf-α*, *nf-kb*, and *il-1β* were markedly higher in the HLM1, HLM5, HLM6 and HLM7, while the gene *iL-10* belonging to anti-inflammatory response was significantly declined in the same groups (*p* < 0.05). The HLM2 and HLM3 groups elevated the expression of *iL-10* in the liver (*p* < 0.05).

### 3.9. Antioxidant and Ferroptosis-Associated Transcriptional Changes in the Liver of T. ovatus

**Figure 11:**
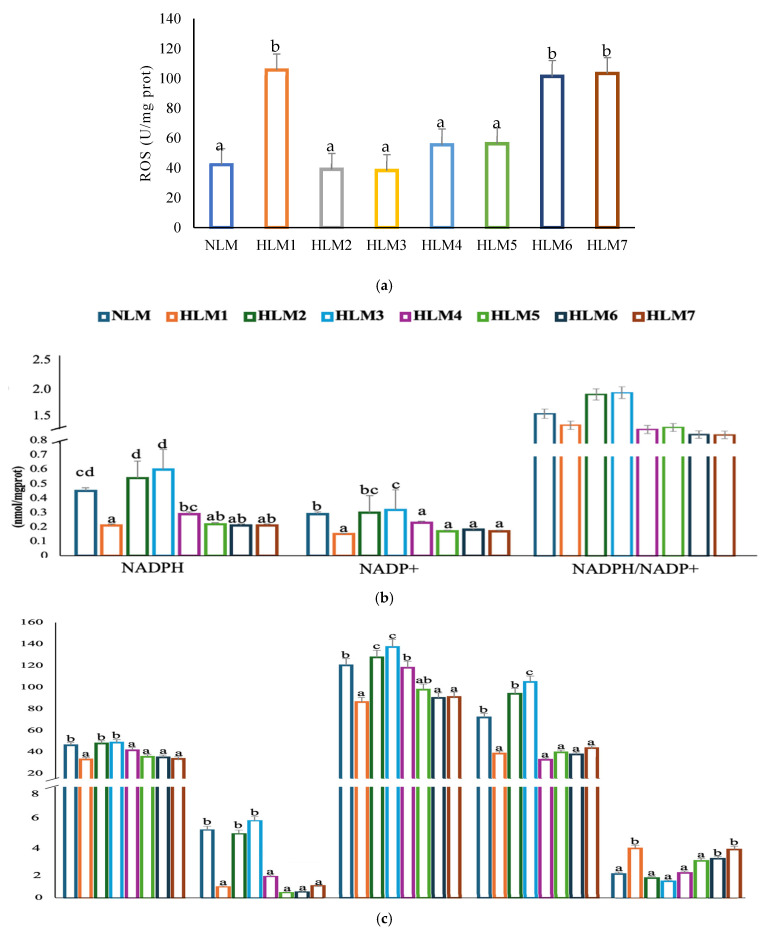

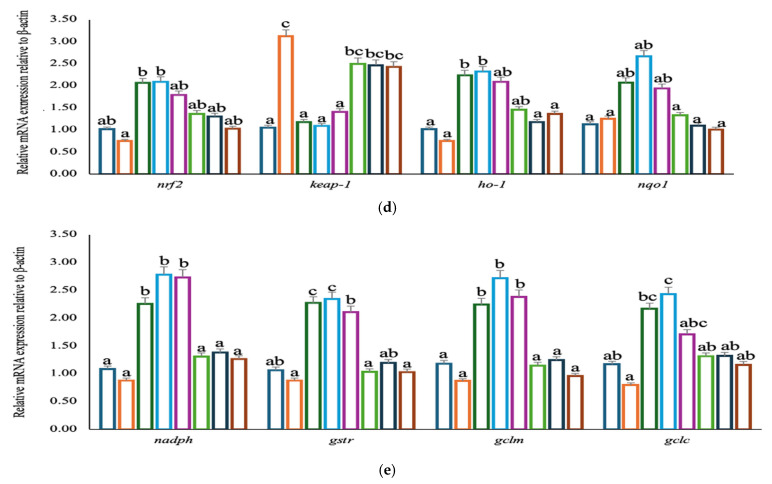
The effect of high-lipid diet and Met on liver antioxidants and oxidative stress in adult *T. ovatus.* (**a**) The content of ROS in the liver of adult *T. ovatus*. (**b**) Hepatic NADPH content of experimental fish, (**c**) oxidative and antioxidant enzymes in the liver, and (**d**) relative mRNA expression levels of *nrf2* signaling pathway genes in the liver of *T. ovatus*. (**e**) Hepatic relative mRNA expression levels of antioxidant signaling genes.

*T. ovatus* fed with HLM1, HLM6, and HLM7 dramatically increased hepatic ROS and MDA levels, while the same parameters were lowered by HLM2 and HLM3 (*p* < 0.05) (Figure 11a,c). Notwithstanding, the antioxidants such as NADPH, NADP^+^, SOD, T-AOC, GSH-px and GSH as well as relative mRNA expression of antioxidant-related genes (*nrf2*, *ho-1*, *nqo1*, *nadph*, *gstr*, *gclc*, and *gclm*) levels were significantly elevated in the liver of *T. ovatus* fed HLM2 and HLM3 compared to HLM1, HLM6 and HLM7 (*p* < 0.05, Figure 11b–e). The relative hepatic mRNA expression levels of *keap-1* in the liver were enhanced considerably by HLM1, HLM6 and HLM7 yet lowered by HLM3 (*p* < 0.05, Figure 11d).

**Figure 12:**
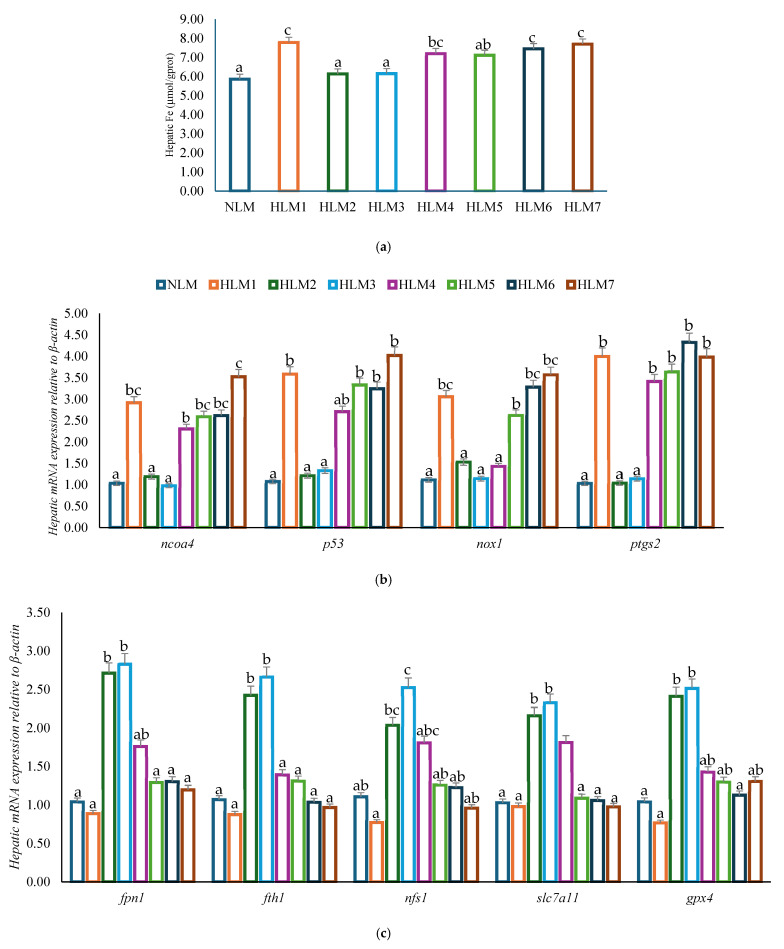
The effect of dietary Met and high lipid on the mRNA expression of ferroptosis-related genes in the liver of adult *T. ovatus*. (**a**) Fe content in the liver of *T. ovatus*. (**b**) Relative mRNA expressions of ferroptosis induction genes. (**c**) Relative hepatic mRNA expressions of anti-ferroptosis-related genes.

Ferroptosis-related genes are presented in (Figure 12a–c). The Fe content in the liver of experimental fish fed with HLM1, HLM6 and HLM7 was significantly high compared to HLM2 and HLM3 (*p* < 0.05, Figure 12a). The relative mRNA expression of ferroptosis promoter genes *ncoa4*, *p53*, *nox1*, and *ptgs2* in fish liver was markedly higher in groups HLM1, HLM6 and HLM7 than those in groups HLM2 and HLM3 (*p* < 0.05, Figure 12b). The genes *fpn1*, *fth1*, *nfs1*, *slc7a11*, and *gpx4* were significantly elevated in the HLM2 and HLM3 groups yet HLM1, HLM5, HLM6 and HLM7 experienced significantly low expression levels (*p* < 0.05, Figure 12c).

**Figure 13:**
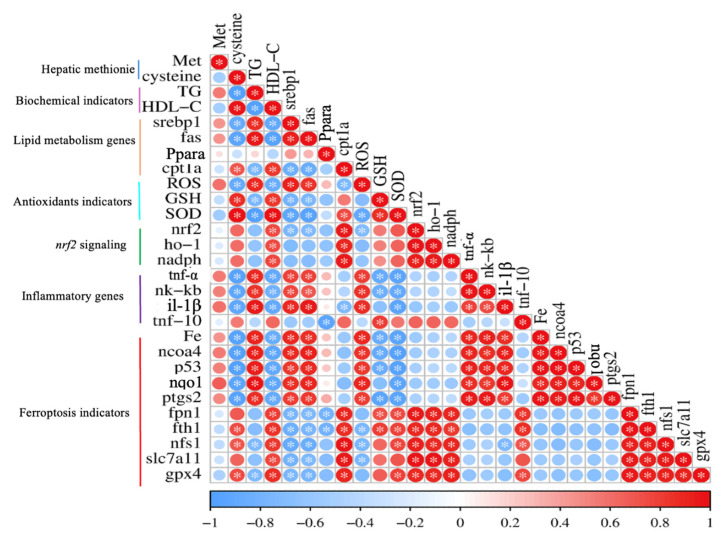
Correlation analysis of hepatic Met, biochemical indicators, antioxidant defense, and the transcription of genes participating in lipid metabolism, *nrf2* signaling, inflammatory and ferroptosis responses. Color depth signifies the magnitude of the correlation, with red indicating a positive correlation and blue indicating a negative correlation. The size of the circle is proportional to the value. An asterisk (∗) denotes a significance level of *p* < 0.05.

The correlation analysis revealed strong and biologically meaningful associations among hepatic amino acid levels, biochemical indicators, antioxidant capacity, lipid metabolism, inflammatory responses, and ferroptosis-related response in *T. ovatus* (Figure 13). The lipolysis-related genes (*Pparα* and *cpt1a*) were negatively correlated with ROS and MDA but positively correlated with antioxidant indicators (GSH, SOD, and CAT) (*p* < 0.05), while the ferroptosis-associated genes (*ncoa4*, *p53*, *nox1*, and *ptgs2*) exhibited strong positive correlations with Fe content, ROS, MDA, and inflammatory markers (*p* < 0.05), indicating that iron accumulation and oxidative stress are closely linked to ferroptotic processes. These genes were also positively associated with the lipogenesis markers (*p* < 0.05, Figure 13). Notably, *gpx4* and *slc7a11* were strongly associated with hepatic cysteine and GSH levels, possibly, highlighting the importance of the glutathione-dependent antioxidant system in suppressing ferroptosis.

## 4. Discussion

### 4.1. Effect of Dietary Met on Growth Performance and Feed Utilization in T. ovatus

Diet with optimum Met enhanced growth across a range of marine fish species, including hybrid grouper (*Epinephelus fuscoguttatus*♀ *× E. lanceolatus*♂) [30], cobia (*Rachycentron canadum*) [31], *S. maximus L.* [32], *D. labrax* [33], *T. ovatus* [34], and *T. blochii* [35]. Supplemented Met in HLDs of juvenile black seabream and juvenile yellow croaker had no effect on growth performance or feed utilization [13,14]. Meanwhile, Met deficiency in HLDs retarded growth in hybrid grouper [36]. In the current study, the inclusion of 1.24% and 1.34% Met in the HLD (HLM3 and HLM4) improved the growth performance and feed utilization of adult *T. ovatus*, whereas higher and lower inclusion levels of Met in HLDs did not enhance growth performance or feed utilization. The effect of Met on growth performance of fish fed HLD showed a dose-dependent pattern.

### 4.2. Effect of Dietary Met on Lipid Metabolism in T. ovatus Fed HLD in Serum and Hepatic Indices

Met is known as a signaling molecule that regulates lipid metabolism [37]. The changes in T-CHO and TG concentrations in the serum emanates from the breakdown of lipids and proteins, and these levels can help to identify changes in metabolism [38,39,40]. However, high HDL-C levels have been associated with healthy lipid metabolism and cardiovascular/liver health [41,42,43]. Higher serum LDL-C and NEFAs levels indicate hepatic lipid buildup, metabolic syndrome, or a fatty liver [44,45]. Once the liver is injured by oxidative stress, liver cell (hepatocyte) membranes degrade, allowing ALT and AST to escape into the bloodstream [46,47,48]. In the current study, serum T-CHO, TG, LDL-C, AST and ALT in groups HLM1, HLM6 and HLM7 significantly increased compared to NLM, HLM2 and HLM3 as well as NEFA levels. Those hinted the liver of fish in groups HLM1, HLM6 and HLM7 may be injured. And higher HDL-C levels were shown in HLM3 group compared to the other high-lipid groups, demonstrating that fish ingesting diet containing the 18% lipid got liver dysregulated lipid metabolism, but optimal Met level improved lipid metabolism homeostasis of fish in HLM3 group. Moreover, the results regarding the serum biochemical indices were corroborated by the liver H&E and oil red o slice analysis. *T. ovatus* fed with NLM, HLM2 and HLM3 exhibited a significant reduction in lipid droplet area. These current findings are somewhat consistent with prior studies on yellow croaker [14], hybrid grouper [36], juvenile black seabream [13], and rice field eel [37]. Although HSI is often used as an indicator of hepatic enlargement and lipid deposition, it should be interpreted together with growth, biochemical, histological, and molecular indicators. In growing rockfish (*Sebastes schlegeli*), increased dietary lipid significantly elevated HSI, but the fish growth performance and feed utilization were improved [17]. Similarly, in the present study, the higher HSI observed in HLM3 did not appear to indicate hepatic injury, because this group showed improved growth performance, reduced serum T-CHO, TG, LDL-C, AST, and ALT compared with the high-Met group, lower hepatic lipid droplet accumulation, and enhanced antioxidant capacity. Thus, the increased HSI in HLM3 may represent adaptive hepatic nutrient metabolism rather than pathological lipid accumulation.

### 4.3. Transcriptomics Analysis of Fish Liver

Based on the growth performance data, NLM, HLM1 and HLM3 were selected to explore transcriptomics analysis. Results presented that the identified DEGs corresponding to biological processes were involved in metabolic processes, immune system processes and growth. And the KEGG enrichment analysis revealed that lipid metabolism, immune system, cell death and growth, transport and catabolism were among the most enriched in DEGs. According to interpretation of the transcriptomic results, hepatic ferroptosis (ko04216), *p53* signaling pathway (ko04115), *ppar* signaling pathway (ko03320), biosynthesis of unsaturated fatty acid (ko01040), and *TNF* signaling pathway (ko04668) were sensitive to the HLDs and dietary Met levels treatments. High lipid accumulation is associated with elevated tissue iron and ferroptosis [49], and dietary Met was the main regulator of lipid metabolism, immunity and antioxidant response [37]. Beyond that, transcriptomic data further hinted that the signaling pathways responsible for lipid metabolism, ferroptosis, and immune responses might be the mechanisms to explain how dietary Met enhanced lipid metabolism and growth in the *T. ovatus* fed HLDs.

#### 4.3.1. Hepatic Lipid Metabolism in *T. ovatus*

In the present study, the fish fed with HLM2 and HLM3 had higher hepatic mRNA expression levels in lipolysis such as *cpt1a*, *ppara*, *atgl*, *hl*, and *lpl* of the adult *T. ovatus.* The lipogenesis-related genes *srebp1*, *fas* and *acc* were markedly upregulated in the HLM1 and HLM7. In previous studies, hepatic *acc*, *fas*, *srebp1*, and *fad* were elevated under higher lipid feeding condition, while *lpl*, *cpt1* and *ppara* were suppressed in juvenile *T. ovatus* [36,50,51]. Also, *ppara* and *cpt-1* were down-regulated in juvenile hybrid grouper fed HLD with low dietary Met inclusion [36,50]. The *srebp-1c* and *fas* expression in the liver of juvenile black seabream were elevated by 17%HLD; however, optimal dietary Met inclusion elevated *ppara*, *cpt1a*, *hsl* and *lpl* in the same [13]. The possible reason was that, at the lipid level of experimental feed, the concentration of Met was either too high or too low. Similar phenomena were also observed in the high-Met and low-Met groups of this experiment. The 18% dietary lipid elevated lipogenesis, while optimal dietary Met inclusion activated lipolysis.

#### 4.3.2. Hepatic Antioxidant Defense and Anti-Inflammatory Signaling

The antioxidant defense markers of fish such as SOD, CAT, GSH-Px, GSH, and T-AOC aids to combat oxidative stress and it encompasses enzymatic and non-enzymatic markers [52,53,54,55,56]. In this study, the *T. ovatus* fed HLM1, HLM6 and HLM7 had significantly higher levels of MDA and ROS, while the antioxidant defense markers were significantly reduced. It showed that higher lipid induced the oxidant and that an inappropriate level of Met not only fails to address this situation but also makes it worse. Interestingly, HLM2, HLM3 and HLM4 made the situation better and had high amounts of NADPH.

The *keap1* mRNA expression was found to be high in HLM1 and HLM5-HLM7, and these groups had low levels of *nrf2*, *ho-1*,*nqo1*, *gstr*, *gclm*, and *gclc*. However, elevated levels of *nrf2* and its downstream genes *ho-1*, *nqo1*, *gstr*, *gclm*, and *gclc* were seen in the *T. ovatus* fed HLM2, HLM3 and HLM4. Under oxidative stress, *nrf2* moves to the nucleus and promotes the transcription of these cytoprotective genes [57,58]. This suggests that the HLDs weakened the antioxidant defense of *T. ovatus* via suppressing *nrf2* signaling, while the optimal dietary Met levels in the HLM2-HLM4 enhanced the antioxidant defense. Elevated *ho-1* and *nqo1* promote detoxification, and *gstr*, *gclm*, and *gclc* are involved in glutathione production, which is necessary for neutralizing ROS, which is consistent with a previous study where oxidative stress suppressed *nrf2* and *ho-1* expression; however, optimal Met supplementation enhanced antioxidant defense via elevated *nrf2* and *ho-1* signaling in juvenile blunt snout bream [59], juvenile largemouth bass (*Micropterus salmoides*) [60] and grass carp (*Ctenopharyngodon idella*) [61].

The inflammatory response in fish is orchestrated by innate immune cells [62]. In the present study, the pro-inflammation response indicators (*tnf-α*, *il-1β*, and *nf-kb*) were significantly elevated in the HLM1, HLM6 and HLM7 groups, while the same were suppressed in the HLM2 and HLM3. Interestingly, the *iL-10* showed the opposite trend. Higher lipid ingestion in Japanese seabass (*Lateolabrax japonicus*) resulted in high serum and hepatic lipid overload, which caused hepatic oxidative stress damages, which lead to the activation of damage-associated molecular patterns triggered by inflammation markers (*il-1β*, *tgfβ*, *tnf-α*, and *il8*) in the liver [63]. Dietary Met at optimal level in HLD reduced serum and hepatic lipid levels and inhibited hepatic inflammation signaling in black seabream [13]. These changes indicate that moderate dietary Met in the HLM3 group attenuated hepatic inflammation triggered by the 18% lipid, possibly by the enhanced antioxidant capacity via the *nrf2*/*keap1* response, thereby promoting hepatic homeostasis.

#### 4.3.3. Dietary Met Was Associated with Transcriptional Changes in Hepatic Ferroptosis-Related Genes

Ferroptosis is a type of regulated cell death triggered by iron, characterized by cell membrane damage [64]. Lipid peroxidation exhibited a strong positive correlation with iron accumulation and disrupted antioxidant system homeostasis in fish [49,65,66,67]. One of the key KEGG pathways revealed by the transcriptome sequencing in the current investigation was cell death and growth, which contained substantial DEGs associated with ferroptosis, *gpx4*,*sat1*, *slc40a1* (also known as *fpn1*), and *acsl4*. The hepatic lipid and iron contents were significantly elevated in the HLM1 group and in the HLM6 and HLM7 groups. It suggested that higher dietary lipid intake induced oxidative stress, which, in turn, might result in ferroptosis; although Met was supplied, the Met dose was either too high or too low. However, optimal Met in the HLM2 and HLM3 groups significantly improved the phenomenon. Upregulated *ptgs2*, *ncoa4*, and *nox1* could regulate free iron (Fe^2+^) and produce superoxide (O_2_^−^) to drive lipid oxidation stress and promote ferroptosis [68].

The high-lipid control HLM1 triggered high levels of *ptgs2*, *nox1*, *ncoa4*, and *p53*, while the normal-lipid control NLM and the HLM2 and HLM3 groups reduced these levels. *Fth1* stores iron safely and prevents Fe^2+^ from catalyzing ROS, *fpn1* exports iron out of cells, reduces intracellular iron, and protects cells from hepatic iron accumulation [69], and *nfs1* maintains mitochondrial Fe–S proteins to stabilize iron metabolism and suppresses free iron accumulation [68,69,70]. Interestingly, the *nfs1*, *gpx4*, *slc7a11*, *fth1*, and *fpn1* were significantly elevated in the NLM, HLM2, and HLM3 groups, which had a strong positive correlation with hepatic Met, cysteine, GSH concentration, and *nrf2* expression. *Slc7a11* imports cystine, used to produce glutathione, which maintains antioxidant defense. Met contributes to the transsulfuration pathway, in which it is converted to homocysteine and then to cysteine. This is used to produce GSH, a powerful antioxidant that neutralizes ROS and protects cells from oxidative stress [71,72]. GSH is directly implicated in lipid hydroperoxide reduction via *gpx4*, a central ferroptosis suppressor [73,74]. Prior study indicated that dietary Met via *nrf2* signaling pathway reduced ferroptosis induced by heat stress bovine mammary epithelial cell [75,76].

Reduced Met intake decreases malondialdehyde (MDA) levels in grass carp, while appropriate supplementation enhances glutathione (GSH)-mediated antioxidant capacity and reduces ROS accumulation in black seabream fed high-fat diets [13,77]. These findings indicate that Met imbalance, whether deficient or excessive in aquatic organisms, influences antioxidant defense and lipid oxidation. High-lipid diets are associated with metabolic disorders that promote lipid peroxidation, ROS accumulation, and iron dysregulation, all of which are key drivers of ferroptosis-related cellular injury [22,78]. Our previous study on hybrid grouper under high-lipid dietary conditions showed that Met restriction promoted hepatic lipid accumulation, enhanced lipid oxidation, and increased ROS production, whereas optimal dietary Met supplementation effectively alleviated these effects by improving lipid utilization and redox homeostasis [36]. These physiological changes—particularly excessive lipid deposition and elevated oxidative stress—are key upstream events closely associated with the initiation of ferroptosis. Consistent with this, the present study’s transcriptomic analysis revealed that ferroptosis-related pathways were significantly enriched under high-lipid dietary conditions across NLM, HLM1, and HLM3. Therefore, based on both our previous findings and the current molecular evidence, we hypothesize that dietary Met plays a regulatory role in modulating ferroptosis-related responses in *T. ovatus* under high-lipid dietary stress. In the present study, reduced oxidative stress was observed in HLM2 and HLM3, suggesting that appropriate Met supplementation may suppress HLD-induced ferroptosis-like responses in adult *T. ovatus*. Met could regulate redox homeostasis and the *Nrf2*-mediated antioxidant system.

While high amounts of Met and cysteine help antioxidant systems, an excess of these amino acids can cause an imbalance in the transsulfuration pathway, resulting in excess homocysteine and potentially contributing to oxidative stress [79]. Elevated homocysteine levels have been related to a variety of oxidative and metabolic problems, which, if not managed appropriately, can lead to cellular damage [80]. Furthermore, high-lipid consumption, especially when combined with an imbalance in antioxidant defenses, has the potential to overwhelm the glutathione system, resulting in lipid peroxidation and ferroptosis, particularly in the liver, where lipid and antioxidant metabolism is very active. In fish, lipid peroxidation, iron accumulation, ROS, and MDA are directly linked to ferroptosis [79,81,82]. This could explain why high levels of Met inclusion in groups HLM6 and HLM7 exhibited a strong positive correlation with pro-ferroptosis indicators in this study.

Lipolysis-related genes, including *Pparα* and *cpt1a*, were negatively correlated with ROS and MDA but positively associated with antioxidant indicators (GSH, SOD, and CAT). It indicated that enhanced fatty acid β-oxidation is closely linked to improved redox homeostasis, consistent with previous reports that activation of *Pparα–cpt1a* signaling improves lipid utilization and reduces oxidative stress in fish [13,36]. In contrast, ferroptosis-related genes, including *ncoa4*, *p53*, *nox1*, and *ptgs2*, showed strong positive correlations with hepatic Fe content, ROS, MDA, inflammatory markers, and lipogenesis-related genes, suggesting that iron accumulation and oxidative stress promote ferroptosis-associated hepatic injury, consistent with iron-dependent lipid peroxidation mechanisms in aquatic organisms [83,84]. Notably, *gpx4* and *slc7a11* were positively correlated with hepatic cysteine and GSH levels, highlighting the importance of the glutathione-dependent antioxidant system in suppressing ferroptosis [85]. Overall, these results demonstrated a coordinated interaction among lipid metabolism, redox balance, iron homeostasis, and ferroptosis-associated responses, supporting the role of dietary Met in regulating hepatic metabolic health in *T. ovatus*.

## 5. Limitation Notes

The RNA-seq analysis was performed only in the selected representative groups, namely NLM, HLM1, and HLM3; therefore, the transcriptomic conclusions should be interpreted as applying only to these groups. Based on the groups NLM, HLM1, and HLM3, the transcriptome sequence data revealed that critical associated lipid metabolism, classic antioxidant pathway *Nrf2*, and ferroptosis markers were influenced by Met and higher lipid. And the expression of these genes was further tested in all experimental groups, including HLM6 and HLM7. The interpretation was mainly supported by growth performance, biochemical indices, histology, and targeted gene-expression analysis rather than whole-transcriptome evidence.

## 6. Conclusions

High-lipid diet-induced hepatic injury is likely a complex process involving multiple interacting mechanisms, including oxidative stress, lipid peroxidation, inflammation, and so on. The present study provides an indispensable insight into the utilization of HLDs in aquaculture production. HLD ingestion in *T. ovatus* could disrupt serum biochemical indices and body antioxidant defense and promote ROS and Fe production, lipogenesis, and inflammation, factors which may trigger ferroptosis in the *T. ovatus* liver. Optimal dietary Met was involved in key genes expression on pathways *nrf2*/*keap1*, metabolism and ferroptosis to make fish fed HLD grow well. Quadratic regression analysis estimated the optimal dietary Met requirement to be 1.32%, which could made fish fed HLD get better WGR.

## Figures and Tables

**Table 1:** Composition and nutrient levels of the experimental diets fed to adult *T. ovatus* (dry matter basis, %).

Ingredients	NLM	HLM1	HLM2	HLM3	HLM4	HLM5	HLM6	HLM7
Crude lipid/Met	11/1.04	18/1.04	18/1.14	18/1.24	18/1.34	18/1.44	18/1.54	18/1.64
Brown fish meal	24.00	24.00	24.00	24.00	24.00	24.00	24.00	24.00
Corn gluten flour	10.00	10.00	10.00	10.00	10.00	10.00	10.00	10.00
Soy protein concentrate	16.00	16.00	16.00	16.00	16.00	16.00	16.00	16.00
Cottonseed protein concentrate	9.00	9.00	9.00	9.00	9.00	9.00	9.00	9.00
Peanut meal	5.00	5.00	5.00	5.00	5.00	5.00	5.00	5.00
Wheat flour	18.00	18.00	18.00	18.00	18.00	18.00	18.00	18.00
Fish oil	3.00	6.00	6.00	6.00	6.00	6.00	6.00	6.00
Soybean oil	3.00	6.40	6.40	6.40	6.40	6.40	6.40	6.40
Soybean lecithin	1.50	1.50	1.50	1.50	1.50	1.50	1.50	1.50
Chlorine chloride	0.05	0.05	0.05	0.04	0.04	0.04	0.04	0.04
L-Met ^a^	0.12	0.12	0.22	0.32	0.42	0.52	0.62	0.72
Vitamin C	0.10	0.10	0.10	0.10	0.10	0.10	0.10	0.10
Vitamin premix ^b^	1.00	1.00	1.00	1.00	1.00	1.00	1.00	1.00
Mineral premix ^c^	1.00	1.00	1.00	1.00	1.00	1.00	1.00	1.00
Ethoxyquin	0.03	0.03	0.03	0.03	0.03	0.03	0.03	0.03
Calcium monophosphate	1.20	1.20	1.20	1.20	1.20	1.20	1.20	1.20
Rice brand	7.00	0.60	0.50	0.40	0.30	0.20	0.10	0.00
Total	100.00	100.00	100.00	100.00	100.00	100.00	100.00	100.00
Proximate composition ^d^								
Moisture	13.18	13.27	13.40	13.29	13.29	13.21	13.13	13.18
Crude Protein	41.58	41.56	41.54	42.14	41.44	42.09	41.75	41.53
Crude lipid	11.29	17.91	18.03	18.08	17.81	18.31	18.30	18.10
Met	1.04	1.02	1.10	1.24	1.39	1.47	1.51	1.63

^a^ Dietary L-Met provided by Gunagdong Yuehai Feeds Co., Ltd, Zhanjiang, China. ^b^ Vitamin premix (per kg diet): VA, 1,100,000IU; D3, 320,000IU; VB12, 8 mg; VK3, 1000 mg; VB1, 1500 mg; VB2, 2800 mg; calcium pantothenate, 2000 mg; nicotinamide, 7800 mg; folic acid, 400 mg; inositol, 12,800 mg; VB6, 1000 mg. ^c^ Mineral premix (per kg diet): sodium fluoride, 2 mg; potassium iodide, 0.8 mg; cobalt chloride (1%), 50 mg; copper sulphate, 10 mg; ferrous sulphate, 80 mg; zinc sulphate, 50 mg; manganese sulphate, 60 mg; magnesium sulphate, 1200 mg. ^d^ The proximate composition was measured value.

**Table 2:** Amino acid profile of the experimental diets (dry matter basis, %).

Amino Acids	NLM	HLM1	HLM2	HLM3	HLM4	HLM5	HLM6	HLM7
EAA ^a^								
Met	1.02	1.04	1.11	1.24	1.39	1.45	1.53	1.64
Valine	2.01	1.9	1.88	1.92	1.93	1.92	1.95	1.94
Isoleucine	1.7	1.63	1.61	1.64	1.64	1.63	1.68	1.67
Phenylalanine	1.98	2.01	2.00	2.01	2.01	1.99	1.99	1.98
Leucine	3.31	3.38	3.36	3.36	3.35	3.33	3.34	3.33
Lysine	2.5	2.51	2.49	2.53	2.55	2.53	2.46	2.45
Histidine	1.02	1.04	1.03	1.03	1.04	1.02	1.03	1.02
Arginine	2.91	2.93	2.91	2.93	2.92	2.91	2.92	2.91
Threonine	1.51	1.63	1.62	1.61	1.6	1.58	1.50	1.49
NEAA ^b^								
Tyrosine	1.26	1.38	1.36	1.35	1.31	1.30	1.34	1.33
Alanine	2.19	2.24	2.23	2.22	2.21	2.19	2.21	2.20
Glycine	2.10	2.11	2.09	2.09	2.09	2.07	2.07	2.06
Glutamic acid	7.54	7.63	7.62	7.65	7.67	7.66	7.42	7.42
Serine	1.67	1.88	1.88	1.86	1.86	1.84	1.68	1.68
Cystine	0.45	0.49	0.48	0.45	0.43	0.41	0.43	0.42
Aspartic acid	3.85	3.66	3.45	3.88	3.39	3.49	3.59	3.38
Proline	2.09	2.22	2.21	2.18	2.15	2.14	2.15	2.14
Summation of AA	39.41	39.40	39.20	39.93	39.56	39.76	39.01	38.92

^a^ EAA, essential amino acids; ^b^ NEAA, non-essential amino acids.

**Table 3:** Contents of fatty acids of experimental diets (dry matter basis, %).

Fatty Acid	NLM	HLM1	HLM2	HLM3	HLM4	HLM5	HLM6	HLM7
C12:0	0.42	0.85	0.63	0.67	0.64	0.67	0.71	0.79
C14:0	23.23	37.94	35.59	35.49	35.20	35.24	37.79	36.36
C15:0	2.26	4.18	3.78	3.75	3.70	3.72	4.04	4.00
C16:0	133.8	210.99	204.23	199.99	196.21	195.37	210.85	207.44
C17:0	6.59	10.85	10.28	10.06	9.86	9.89	10.71	10.61
C18:0	31.23	52.81	52.13	51.28	47.77	47.17	52.67	52.89
C20:0	3.03	5.82	5.62	5.6	5.26	5.14	5.67	5.82
C20:1	9.80	14.73	14.77	14.54	12.12	11.93	14.58	14.98
C21:0	0.5	0.74	0.57	0.45	0.64	0.61	0.60	0.72
C20:2	0.95	1.38	1.23	1.54	1.29	1.17	1.24	1.40
C22:0	38.87	61.27	59.02	48.57	57.97	55.70	61.12	60.40
C24:0	1.06	1.89	2.02	2.35	2.17	1.94	1.75	2.04
C14:1n5	0.85	1.52	1.27	0.28	1.18	1.25	1.38	1.45
C16:1n7	25.99	43.56	40.68	41.98	41.29	39.68	43.42	41.82
C17:1n7	4.41	7.20	6.80	6.95	7.44	6.43	7.06	7.10
C18:1n9c	134.23	239.89	234.74	224.86	212.01	211.90	239.75	238.21
C22:1n9	1.63	3.06	2.95	2.62	2.96	2.99	2.92	3.02
C24:1n9	34.33	56.67	54.28	51.68	48.20	51.19	56.52	55.58
C18:3n3	23.92	46.43	44.51	41.76	43.45	42.68	46.29	45.34
C20:3n3	0.46	0.89	0.73	0.64	0.67	0.71	0.75	0.88
C18:2n6c	211.32	371.28	361.89	351.05	345.79	337.08	371.14	366.40
C18:3n6	0.75	1.49	1.23	1.01	1.14	1.21	1.35	1.42
C20:3n6	0.55	1.06	0.87	0.91	1.25	1.16	0.92	1.03
C20:4n6	3.14	5.64	5.22	5.63	5.51	4.98	5.49	5.50
ΣSFA	251.73	403.45	389.86	374.28	372.82	368.55	401.73	397.46
ΣMUFA	201.44	351.91	340.72	328.38	313.08	313.45	351.05	347.18
Σn-3 PUFA	24.37	47.32	45.24	42.4	44.12	43.39	47.04	46.22
Σn-6 PUFA	215.76	379.48	369.21	358.59	353.69	344.43	378.90	374.35

ΣSFA, sum of saturated fatty acids; ΣMUFA, sum of monounsaturated fatty acids; Σn-3 PUFA, sum of n-3 polyunsaturated fatty acids; Σn-6 PUFA, sum of n-6 polyunsaturated fatty acids.

**Table 4:** Effect of dietary Met inclusions on the growth indices of *T. ovatus* fed HLDS.

Index	Experimental Groups							
	NLM	HLM1	HLM2	HLM3	HLM4	HLM5	HLM6	HLM7
IBW ^1^	82.34 ±0.04	82.42 ± 0.04	82.42 ± 0.04	82.42 ± 0.04	82.44 ± 0.04	82.38 ± 0.00	82.46 ± 0.04	82.44 ± 0.03
FBW ^2^	219.59 ± 14.22 ^ab^	175.60 ± 010.10 ^a^	230.30 ± 11.52 ^ab^	263.17 ± 23.35 ^b^	235.88 ± 6.88 ^ab^	212.17 ± 6.76 ^ab^	199.30 ± 15.23 ^a^	182.23 ± 1.43 ^a^
SGR ^3^	1.74 ± 0.12 ^abc^	1.35 ± 0.10 ^a^	1.83 ± 0.0 ^abc^	2.06 ± 0.17 ^c^	1.88 ± 0.05 ^bc^	1.69 ± 0.06 ^abc^	1.56 ± 0.14 ^abc^	1.41 ± 0.01 ^ab^
WGR ^4^	166.71 ± 17.27 ^ab^	113.07 ± 12.31 ^a^	180.95 ± 13.02 ^ab^	219.30 ± 28.25 ^b^	186.14 ± 8.22 ^ab^	157.56 ± 8.20 ^ab^	141.71 ± 18.58 ^a^	121.07 ± 1.83 ^a^
FE ^5^	72.02 ± 7.48 ^ab^	48.98 ± 5.29 ^a^	77.87 ± 6.20 ^ab^	95.04 ± 12.28 ^b^	80.68 ± 3.62 ^ab^	68.18 ± 3.52 ^ab^	61.31 ± 7.99 ^a^	52.41 ± 0.77 ^a^
HIS ^6^	1.44 ± 0.09 ^abc^	1.16 ± 0.01 ^a^	1.39 ± 0.06 ^abc^	1.81 ± 0.18 ^c^	1.75 ± 0.04 ^c^	1.68 ± 0.10 ^bc^	1.64 ± 0.07 ^bc^	1.26 ± 0.04 ^ab^
PER ^7^	2.81 ± 0.29 ^ab^	1.95 ± 0.21 ^a^	2.71 ± 0.21 ^ab^	3.30 ± 0.43 ^b^	2.84 ± 0.13 ^ab^	2.44 ± 0.14 ^ab^	2.16 ± 0.28 ^ab^	1.87 ± 0.04 ^a^
LER ^8^	4.52 ± 0.27 ^cd^	2.10 ± 0.33 ^a^	4.73 ± 0.70 ^cd^	5.92 ± 0.86 ^d^	4.03 ± 0.16 ^bc^	3.50 ± 0.21 ^abc^	2.95 ± 0.37 ^abc^	2.51 ± 0.01 ^ab^
VSI ^9^	6.33 ± 0.28	5.79 ± 0.13	6.34 ± 0.18	6.45 ± 0.26	6.11 ± 0.12	6.20 ± 0.29	5.93 ± 0.34	5.71 ± 0.18
CF ^10^	3.59 ± 0.01	3.33 ± 0.04	3.54 ± 0.09	3.87 ± 0.23	3.55 ± 0.10	3.36 ± 0.11	3.32 ± 0.33	3.25 ± 0.21

^1^. IBW (g): initial body weight; ^2^. FBW (g): final body weight; ^3^. SGR (%day^−1^): specific growth rate; ^4^. WGR (%): weight gain rate; ^5^. FE: feed efficiency, ^6^. HIS (%), hepatosomatic index, ^7^. PER: protein efficiency ratio; ^8^. LER: lipid efficiency ratio; ^9^. VSI (%): viscerosomatic index, ^10^. CF (g/cm^3^): condition factor. Data represent the means with SEM of three replicates. Values within the same row with different superscripts are significantly different (*p* < 0.05) by one-way ANOVA same as the other data representations.

**Table 5:** Proximate composition of *T. ovatus* (%).

Indices	Experimental Groups							
	NLM	HLM1	HLM2	HLM3	HLM4	HLM5	HLM6	HLM7
Moisture	67.46 ± 0.58	63.36 ± 1.00	66.01 ± 1.03	65.90 ± 0.26	64.70 ± 0.88	65.48 ± 1.42	64.54 ± 0.62	63.32 ± 1.50
Crude Protein	48.79 ± 0.00 ^b^	47.69 ± 0.16 ^a^	54.59 ± 0.01 ^d^	54.85 ± 0.15 ^d^	54.04 ± 0.03 ^cd^	53.23 ± 0.42 ^c^	54.08 ± 0.12 ^cd^	53.39 ± 0.31 ^c^
Crude lipid	30.20 ± 1.37 ^a^	45.20 ± 3.26 ^b^	32.79 ± 5.47 ^a^	30.71 ± 0.64 ^a^	38.07 ± 0.28 ^ab^	37.16 ± 0.31 ^ab^	39.60 ± 0.54 ^ab^	39.78 ± 0.42 ^ab^

Values within the same row with different superscripts are significantly different (*p* < 0.05) by one-way ANOVA, same as the other data representations.

**Table 6:** Serum biochemical parameters of adult *T. ovatus* fed HLDs with varied dietary Met levels.

Indices	Experimental Fish							
	NLM	HLM1	HLM2	HLM3	HLM4	HLM5	HLM6	HLM7
T-CHO (mmol/L)	4.81 ± 0.37 ^a^	8.17 ± 0.37 ^c^	5.56 ± 0.15 ^ab^	5.06 ± 0.28 ^a^	5.49 ± 0.51 ^ab^	7.50 ± 0.52 ^bc^	7.71 ± 0.60 ^bc^	7.87 ± 0.66 ^c^
TG (mmol/L)	4.48 ± 0.38 ^a^	8.17 ± 0.29 ^c^	4.84 ± 0.11 ^ab^	4.65 ± 0.29 ^ab^	5.31 ± 0.67 ^abc^	7.54 ± 0.07 ^bc^	7.58 ± 1.35 ^bc^	7.91 ± 0.54 ^c^
LDL-C (mmol/L)	6.26 ± 0.91 ^a^	9.14 ± 0.05 ^b^	6.13 ± 0.98 ^a^	5.73 ± 0.05 ^a^	6.17 ± 0.25 ^b^	9.08 ± 0.02 ^b^	9.08 ± 0.49 ^b^	9.14 ± 0.00 ^b^
HDL-C (mmol/L)	1.80 ± 0.03 ^c^	1.38 ± 0.01 ^a^	1.93 ± 0.16 ^c^	2.02 ± 0.04 ^c^	1.78 ± 0.04 ^bc^	1.45 ± 0.06 ^a^	1.46 ± 0.06 ^ab^	1.45 ± 0.04 ^a^
ALT (U/L)	63.35 ± 3.99 ^a^	88.00 ± 3.06 ^b^	56.11 ± 6.78 ^a^	50.45 ± 3.63 ^a^	63.40 ± 0.80 ^a^	84.54 ± 5.90 ^b^	84.61 ± 3.19 ^b^	84.86 ± 3.16 ^b^
AST (U/L)	57.73 ± 1.36 ^a^	94.38 ± 0.88 ^b^	63.26 ± 4.34 ^a^	58.09 ± 0.15 ^a^	57.24 ± 0.76 ^a^	81.88 ± 2.60 ^b^	84.14 ± 3.67 ^b^	88.25 ± 4.42 ^b^
NEFA (mmol/L)	35.79 ± 0.43 ^a^	62.79 ± 0.34 ^e^	41.15 ± 0.81 ^bc^	38.35 ± 0.12 ^ab^	53.78 ± 1.27 ^d^	44.33 ± 0.72 ^c^	62.06 ± 0.54 ^e^	63.59 ± 0.88 ^e^

T-CHO, total cholesterol; TG, triglycerides; LDL-C, low-density lipoprotein cholesterol; HDL-C, high-density lipoprotein cholesterol; ALT, alanine aminotransferase; AST, aspartate aminotransferase. Values within the same row with different superscripts are significantly different (*p* < 0.05) by one-way ANOVA, same as the other data representations.

**Table 7:** Serum antioxidant and biochemical parameters of adult *T. ovatus* fed HLDs with varied dietary Met levels.

Indices	Experimental Fish							
	NLM	HLM1	HLM2	HLM3	HLM4	HLM5	HLM6	HLM7
SOD (U/mL)	244.47 ± 4.62 ^c^	187.49 ± 2.18 ^a^	236.07 ± 16.40 ^c^	231.81 ± 5.98 ^bc^	200.36 ± 1.60 ^ab^	199.57 ± 8.56 ^ab^	190.13 ± 2.83 ^a^	190.13 ± 1.84 ^a^
CAT (U/mL)	75.93 ± 10.94 ^b^	19.48 ± 0.66 ^a^	83.61 ± 2.19 ^b^	88.80 ± 6.61 ^b^	83.56 ± 0.35 ^b^	78.50 ± 9.94 ^b^	22.32 ± 4.22 ^a^	30.24 ± 0.23 ^a^
GSH-Px (U/mL)	470.09 ± 37.33 ^b^	209.73 ± 31.82 ^a^	577.24 ± 20.33 ^bc^	619.58 ± 11.36 ^c^	265.62 ± 32.72 ^a^	266.48 ± 27.62 ^a^	234.54 ± 18.40 ^a^	189.84 ± 2.47 ^a^
IgM (g/L)	14.04 ± 2.42 ^b^	2.63 ± 0.58 ^a^	15.13 ± 0.22 ^b^	16.20 ± 1.20 ^b^	14.09 ± 1.24 ^b^	5.32 ± 0.01 ^a^	3.28 ± 0.80 ^a^	3.52 ± 0.61 ^a^
MDA (nmol/mL)	6.22 ± 0.07 ^a^	9.94 ± 0.06 ^c^	6.06 ± 0.05 ^a^	5.08 ± 0.61 ^a^	6.42 ± 0.17 ^b^	8.90 ± 0.11 ^b^	9.06 ± 0.03 ^b^	9.31 ± 0.22 ^bc^
T-AOC (U/mL)	12.95 ± 0.09 ^b^	7.80 ± 0.16 ^a^	12.95 ± 0.12 ^b^	13.15 ± 0.35 ^b^	12.12 ± 0.23 ^b^	8.28 ± 0.39 ^a^	8.02 ± 0.72 ^a^	8.31 ± 0.22 ^a^

SOD, superoxide dismutase; CAT, catalase; GSH-Px, glutathione peroxidase; IgM, immunoglobulin M; MDA, malondialdehyde; T-AOC, total antioxidant capacity. Values within the same row with different superscripts are significantly different (*p* < 0.05) by one-way ANOVA, same as the other data representations.

**Table 8:** Hepatic antioxidant and biochemical parameters of adult *T. ovatus* fed HLDs with varied dietary Met levels.

Indices	Experimental Fish							
	NLM	HLM1	HLM2	HLM3	HLM4	HLM5	HLM6	HLM7
SOD (U/mg prot)	48.39 ± 1.17 ^b^	35.88 ± 1.28 ^a^	49.89 ± 2.53 ^b^	50.67 ± 1.50 ^b^	43.97 ± 1.49 ^ab^	38.12 ± 2.32 ^a^	37.8 ± 2.34 ^a^	36.38 ± 1.58 ^a^
CAT (U/mg prot)	13.91 ± 0.31 ^b^	5.01 ± 0.50 ^a^	16.35 ± 0.85 ^b^	17.89 ± 0.09 ^b^	15.71 ± 1.13 ^b^	7.08 ± 1.74 ^a^	6.51 ± 0.96 ^a^	6.88 ± 1.85 ^a^
GSH-Px (U/mg prot)	118.75 ± 9.20 ^bc^	86.24 ± 3.39 ^a^	125.61 ± 8.08 ^c^	134.76 ± 3.04 ^c^	116.46 ± 1.84 ^bc^	97.31 ± 0.59 ^ab^	90.04 ± 0.15 ^a^	90.72 ± 1.36 ^a^
IgM (μg/mg prot)	3.00 ± 0.37 ^b^	0.79 ± 0.26 ^a^	3.11 ± 0.26 ^b^	3.68 ± 0.07 ^b^	0.82 ± 0.29 ^a^	0.66 ± 0.21 ^a^	1.14 ± 0.02 ^a^	0.58 ± 0.03 ^a^
MDA (nmol/mg prot)	1.76 ± 0.20 ^a^	3.59 ± 0.16 ^b^	1.56 ± 0.01 ^a^	1.16 ± 0.29 ^a^	1.9 ± 0.10 ^a^	2.78 ± 0.01 ^b^	2.88 ± 0.26 ^b^	2.93 ± 0.08 ^b^
T-AOC (U/mg prot)	5.29 ± 0.15 ^b^	1.38 ± 0.30 ^a^	5.04 ± 0.34 ^b^	5.93 ± 0.45 ^b^	2.09 ± 0.34 ^a^	0.98 ± 0.08 ^a^	1.03 ± 0.12 ^a^	1.46 ± 0.44 ^a^

Values within the same row with different superscripts are significantly different (*p* < 0.05) by one-way ANOVA, same as the other data representations.

## Data Availability

The raw RNA-seq reads generated in this study have been deposited in the NCBI Sequence Read Archive under BioProject accession number PRJNA1482782. Other data supporting the findings of this study are available from the corresponding author upon reasonable request.

## Supplementary Materials

The following supporting information can be downloaded at: https://www.mdpi.com/article/10.3390/antiox15070873/s1, Table S1: Primers used for lipid metabolism gene expression and validation of DEGs by qRT-PCR.
