# Supplementary material for: Dietary Methionine Affects Lipid Metabolism and Ferroptosis-Related Responses to Modulate Oxidative Stress Induced by High-Lipid-Diet in Golden Pompano (Trachinotus ovatus)
Source: Antioxidants (Basel). 2026 Jul 14;15(7):873. doi: 10.3390/antiox15070873 (PMC13405565; doi:10.3390/antiox15070873)
Supplement: Supplementary file 1 [file antioxidants-15-00873-s001.zip › antioxidants-4345272-supplementary.pdf]

Table S1. Primers used for lipid metabolism gene expression and validation of DEGs by qRT-PCR.

Genes	Forward (5′-3′)	Reverse (5′-3′)	Reference
cpt1a	CTTTAGCCAAGCCCTTCATC	TTAACCAGTCCCGGTGTTTC	KP987456.1
fpn1	CGTCCAGAACAGTTGCGTCATC	AGTTGTCAGAATCCATCCATTGTAGAG	OM643386
ppara	AATCTCAGCGTGTCGTCTT	GGAAATGCTTCGGATACTTG	KP893147
il-10	CGTCCTGGCTCTCTTGTCTCTCCTC	TGTCCATGTCATTGTTTGCCTCATA	KY231908
nrf2	TTGCCTGGACACAACTGCTGTTAC	TCTGTGACGGTGGCAGTGGAC	[55]
keap1	CAGATAGACAGCGTGGTGAAGGC	GACAGTGAGACAGGTTGAAGAACTCC	[55]
ho1	AGAAGATTCAGACAGCAGCAGAACAG	TCATACAGCGAGCACAGGAGGAG	[55]
nqo1	TGGTCCAGGTGTCACGTCTTCC	GACTTGGCGGTGTAGTGCTTGG	[64]
gstr	TTGCCGCCTGTCTGTATCTG	TTCTTGAGCGTGTCTGGACC	MK614713.1
nadph	ATATGTCTGTGCCCAACTGCT	ATCTGCCAGCGGTGTGTATT	AB192470.1
srebp1	GAGCCAAGACAGAGGAGTGT	GTCCTCTTGTCTCCCAGCTT	[8]
acc	GTTGTCAATCCCAGCCGATC	ATCCACAATGTAGGCCCCAA	[39]
fas	GATGGATACAAAGAGCAAGG	GTGGAGCCGATAAGAAGA	[11]
lpl	TTTGTCCTTCCTCGTCACCA	AAGACAGCATCCTCTCCACC	[39]
atgl	CGAAACGGTTATGGATGCCC	TGGACTTGAACTCGGACACG	[11]
ncoa4	AAGATCGACGAATCAGAGTTCACCATC	GTCTGACACAGTCTCCATCGCATC	[64]
p53	ACAACCACTCAGATCAATCGCTTCC	GGAGCCAAGACAATGACAGCCTAC	[64]
nox1	GCGTGCCAGCTCAACTCAGG	AGGGACAGACAGAGCCACAGTG	[64]
ptgs2	TGTCCCGTGGCGTCCTTCC	CCTGAGTGAGACGTGCTTGAGTTG	[64]
gpx4	GACAGATATTGATGGCAACGTGGTTTC	GCTGCTGGTGGACTTGTGATGG	[64]
hl	GTGGTCGGTGGATGGTATGA	TGCCAATGGTGCGGGTT	[44]
slc7a11	GCTAGACCACTACAGTCCATCACAAC	TGCTGCAACACTTCCATCACTCTG	[64]
cyp7a1	TCAAATAGCCAGCGGCAAAC	CCATGACAGCTTCGACCCTC	XM_035635553.1
nfs1	ACCTCCCAGTCCAGCAGAACG	TGTGTGACCTGTGTGTAACTGTGTG	[64]
fth1	CCGCCGCCGCAAGTGTG	TGACAGACCCAGGACTATTGTGAGG	[64]
sat1	CGGATTCGGTGACCATCCAT	AGCCTTAGTCTCTCACTCCCAT	GU588622.1
fads2	GAACAATCCCACTTCAACG	AGGAATCCCATACTTCTCACA	[50]
slc40a1	CGTCCAGAACAGTTGCGTCATC	AGTTGTCAGAATCCATCCATTGTAGAG	[64]
tnf-a	CGCAATCGTAAAGAGTCCCA	AAGTCACAGTCGGCGAAATG	[64]
il-1b	GCGATTTGGTGCGATTTCT	CTCTACTGGCTTGTTGTCTTGC	[64]
faxdc2	AATAGAAGCCAAACTCCTCG;	GGTGCTCGCTAACCAGACAG	[64]
nf-kb	TGCGACAAAGTCCAGAAAGAT	CTGAGGGTGGTAGGTGAAGGG	[61]
acsl4	AGGCAAGGACACGCTGGATAAG	TCCAGTTCATTGTAGGACAGCCA	PRJNA574781
ugt2a1	TGGCGATACACAGGCGAGAA	GTCAGGCTGATCCGCAAACA	[64]
alox5	TCCACCAGACAGTCACACACCTTC	GCCACTCCAAACACCTCCGAGAC	NM_001139832.1
gclm	ATCCTGACTCCAGCGGTTTC	AGAGTCTTGGCGTGGTTCAG	XM_029145961.3
fabp3	TGGGTGTGGGCTTTGCTA	CTTAATGGTGCTCTGGGTCTT	[11]
gclc	GGGCTGATCCCCATGTTGAA	CCAAACTTGGTCTTGCGCTC	XM_047812721.1
β-actin	TACGAGCTGCCTGACGGACA	GGCTGTGATCTCCTTCTGC	KX987228

cpt1a, carnitine palmitoyltransferase 1A; *fpn1*, ferroportin 1; *ppara*, peroxisome proliferator-activated receptor alpha; *il-10*, interleukin 10; *nrf2*, nuclear factor erythroid 2-related factor 2; *keap1*, Kelch-like ECH-associated protein 1; *ho1*, heme oxygenase 1; *nqo1*, NAD(P)H quinone dehydrogenase 1; *gstr*, glutathione S-transferase rho; *nadph*, nicotinamide adenine dinucleotide phosphate, reduced form; *srebp1*,

sterol regulatory element-binding protein 1; *acc*, acetyl-CoA carboxylase; *fas*, fatty acid synthase; *lpl*, lipoprotein lipase; *atgl*, adipose triglyceride lipase; *ncoa4*, nuclear receptor coactivator 4; *p53*, tumor protein p53; *nox1*, NADPH oxidase 1; *ptgs2*, prostaglandin-endoperoxide synthase 2; *gpx4*, glutathione peroxidase 4; *hl*, hepatic lipase; s*lc7a11*, solute carrier family 7 member 11; *cyp7a1*, cholesterol 7 alpha-hydroxylase; *nfs1*, NFS1 cysteine desulfurase; *fth1*, ferritin heavy chain 1; *sat1*, spermidine/spermine N1-acetyltransferase 1; *fads2*, fatty acid desaturase 2; *slc40a1*, solute carrier family 40 member 1; *tnf-α*, tumor necrosis factor alpha; *il-1β*, interleukin 1 beta; *faxdc2*, fatty acid hydroxylase domain containing 2; *nf-κb*, nuclear factor kappa B; *acsl4*, acyl-CoA synthetase long-chain family member 4; *ugt2a1*, UDP glucuronosyltransferase family 2 member A1; *alox5*, arachidonate 5-lipoxygenase; gclm, glutamate-cysteine ligase modifier subunit; *fabp3*, fatty acid-binding protein 3; *gclc*, glutamate-cysteine ligase catalytic subunit; *β-actin*, beta-actin.
